# Drug Tolerant Anti-drug Antibody Assay for Infliximab Treatment in Clinical Practice Identifies Positive Cases Earlier

**DOI:** 10.3389/fimmu.2020.01365

**Published:** 2020-07-21

**Authors:** Nastya Kharlamova, Christina Hermanrud, Nicky Dunn, Malin Ryner, Karen Hambardzumyan, Nancy Vivar Pomiano, Per Marits, Inger Gjertsson, Saedis Saevarsdottir, Rille Pullerits, Anna Fogdell-Hahn

**Affiliations:** ^1^Department of Clinical Neuroscience, Karolinska Institutet, Stockholm, Sweden; ^2^Center for Molecular Medicine, Stockholm, Sweden; ^3^Rheumatology Division, Department of Medicine, Solna, Karolinska Institutet, Stockholm, Sweden; ^4^Department of Clinical Immunology and Transfusion Medicine, Karolinska University Hospital, Stockholm, Sweden; ^5^Department of Rheumatology and Inflammation Research, Institution of Medicine, Sahlgrenska Academy, University of Gothenburg, Gothenburg, Sweden; ^6^Faculty of Medicine, School of Health Sciences, University of Iceland, Reykjavik, Iceland; ^7^Department of Clinical Immunology and Transfusion Medicine, Sahlgrenska University Hospital, Gothenburg, Sweden

**Keywords:** anti-drug antibody, serum infliximab, clinical threshold value, clinical effect, PandA

## Abstract

A subgroup of patients treated with infliximab lose response to the treatment and one reason for this is the development of anti-drug antibodies (ADA). If used optimally, measuring drug and ADA level could lead to a more personalized and efficient treatment regime, and enable identification of ADA-positive patients before the underlying disease flares or allergic reactions occur. With the use of a drug-tolerant ADA assay which can detect ADA irrespective of drug levels in the sample, we determined the impact of ADA on treatment failure to infliximab. The aims of this study were to estimate the real-life optimal serum infliximab (sIFX) level and set a clinical threshold value for a drug-tolerant ADA assay. Trough levels of sIFX were measured with ELISA. Free ADA was measured with two drug-sensitive methods (ELISA and a bioassay) and one drug-tolerant method (PandA). Two real-life cohorts treated with infliximab were included; a cross-sectional cohort including patients with inflammatory rheumatic diseases (*n* = 270) and a prospective cohort of rheumatoid arthritis (RA) patients (*n* = 73) followed for 1 year. Normal range of sIFX was estimated from the prospective cohort and an arbitrary optimal drug level was set to be between 1 and 6 μg/mL. Using this range, optimal sIFX was found in only 60% (163/270) of the patients in the cross-sectional cohort. These patients had significantly better treatment response than those with a drug level under 1 μg/mL, who had an ADA frequency of 34% (19/56) using the drug-tolerant method. In the prospective cohort, the drug-tolerant assay could identify 34% (53/155 samples) as ADA positive in samples with sIFX level >0.2 μg/mL. ADA were seldom detected in patients with >1 μg/mL sIFX, with three interesting exceptions. A clinically relevant ADA threshold was determined to be >3 RECL as measured with the drug-tolerant assay. In a real-life setting, there was a substantial number of patients with suboptimal drug levels and a proportion of these had ADA. Both too low and too high drug levels correlated with worse disease, but for different reasons. Adding a drug-tolerant assay enabled detection of ADA earlier and regardless of drug level at time of sampling.

## Introduction

Infliximab is a chimeric monoclonal antibody (mAb) blocking the effect of tumor necrosis factor alpha (TNF-α) which has been widely used since 1999 for treatment of a number of inflammatory rheumatic diseases including rheumatoid arthritis (RA). TNF-α inhibitors (TNFi) were the first monoclonal antibody therapy shown to significantly halt progression of these diseases in clinical trials (1–3), and the treatment effect is even more efficient in combination with other disease modifying anti-rheumatic drugs (DMARDs) such as methotrexate (MTX) (3). However, up to 40% of patients do not respond to TNFi treatment according to the EULAR (European League Against Rheumatism) response criteria (4–7). These patients can be categorized into those who never achieve any response (primary treatment failure), and those who lose response over time (secondary treatment failure) (8). One cause for secondary treatment failure is the development of anti-drug antibodies (ADA) (9–12). ADA results in reduced availability of the drug in the circulation (13) and therefore a lower effective dose (14–16). There is an association between non-responders and low serum trough infliximab (sIFX) levels (17) that is often due to the development of ADA (18). Therefore, it is recommended to start by first screening for the drug trough level and then usually only those with low drug level are subsequently tested for ADA.

The presence of ADA can lead to a subtherapeutic serum drug level by either neutralization of the drug, leading to hampered pharmacological activity, or through the sequestering of drug resulting in increased clearance of immune complexes (IC) via excretion through the kidneys (19, 20). ADA have also been associated with adverse effects with an increased risk of infusion-reactions, lupus, and vasculitis like events (21). Given that several studies have shown that up to 44% of patients treated with TNFi develop ADA, it is an important clinical issue to address. Routine ADA testing would allow early identification of these patients ensuring an efficient treatment regimen (3, 11, 22).

The prevalence of ADA to TNFi vary between studies, which in part can be explained by differences in concomitant medication use, timing of sampling in relation to the drug administration, treatment duration, and type of assay used for ADA detection (23–25). Standard immunoassays such as ELISA are frequently used for ADA screening. However, disadvantages of these assays include a low drug tolerance and ability to detect only free ADA. When ADA bind to the drug to form immune complexes, the antibodies become indiscernible using standard laboratory techniques, leading to a false negative result (26). One way to overcome the problem of drug interference is to use a drug tolerant, precipitation, and acid dissociation (PandA) assay. This procedure involves the addition of excess drug to the sample followed by dissociation of ADA bound to the drug before detection, making it possible to detect both free and bound ADA in samples regardless of the level of drug in the serum (27).

At times, measurement of trough TNFi level and ADA are used to monitor patients with chronic inflammatory disease when the patient has no or little clinical improvement with treatment (28). However, adjustments of dose and intervals are often made without these types of supporting data. Despite several suggestions, there is no consensus on which ADA detection assay should be used, nor is the optimal drug trough level known. Furthermore, the outcome of ADA and drug level testing varies between different methods (25). In addition, there is a lack of knowledge about how prevalent ADA are in patients with detectable drug trough levels and at what level ADA have a clinically relevant impact (29).

## Materials and Methods

### Patient Cohorts

This study included two cohorts; With patients from (1) a cross-sectional cohort from the Karolinska University Hospital, Stockholm (*n* = 270) and (2) a prospective cohort from the Sahlgrenska University Hospital, Gothenburg (*n* = 73), described in Table 1. In the cross-sectional study, all patients treated with infliximab in the rheumatology clinic between January 2017 to December 2017 were recruited (*n* = 270). Several samples were collected per patient at trough prior to an infusion. In the cross-sectional cohort, 43% (*n* = 115) of the included patients had RA, 44% (*n* = 118) had other type of inflammatory arthritis (spondylarthritis, ankylosing spondylitis, psoriatic arthritis, reactive arthritis, enteropathic arthritis, or undifferentiated) and 14% (*n* = 37) had other systemic inflammatory diseases. All patients (except four) in the cross-sectional cohort were switched to infliximab biosimilar Inflectra^TM^ in 2017. A total of 63% (169/270) of the patients were concomitantly treated with conventional synthetic disease-modifying anti-rheumatic drug (csDMARD) (156 with methotrexate; 6 with sulfasalazine; 4 with azathioprine; 3 with leflunomide).

**Table 1:** Baseline patient characteristics in prospective and cross-sectional cohorts.

**Characteristic**	**Cross-sectional cohort**			**Prospective cohort**
	**RA**	**Spondyloarthropathies**	**Other systemic inflammatory diseases**	**RA**
*n* (%)^*^	115 (42.6)	118 (43.7)	37 (13.7)	73 (100)
Age (median, min-max)	65 (31–83)	51 (20–80)	45 (20–84)	52 (18–89)
Female (*n*,%)	90 (78)	40 (34)	24 (65)	58 (79)
Concomitant DMARD (*n*,%)	91 (79)	54 (46)	24 (65)	72 (98)
MTX (*n*,%)	86 (75)	49 (42)	21 (57)	68 (93)
Other DMARD (*n*,%)	5 (4)	5 (4)	3 (8)	4 (5)
lnfliximab dose (median mg/IV, IQR)	210 (200–300)	250 (200–300)	260 (200–300)	200 (150–200)
Duration of treatment (median, IQR)	9 (4–18)	6 (2–11)	4 (1.5–8)	n.d.
Current smokers (*n*,%)	9 (8)	13 (11)	3 (8)	9 (12.5)
Ever smokers (*n*,%)	61(53)	63 (53)	28 (54)	40 (56)
Never smokers (*n*,%)	53 (46)	53 (45)	17 (46)	32 (44)
Disease duration (median, IQR)	17 (11–23.5)	14 (6.75–24)	11 (4.5–20)	1.5 (0.5–10.55)
Seropositive (*n*,%)	83 (72)	n.d.	n.d.	53 (75)
Seronegative (*n*,%)	32 (28)	n.d.	n.d.	17 (24)
CRP (median mg/L, IQR)	1 (1–4)	1 (1–4)	2 (1–4.5)	4 (1–9.75)
Patient global health assessment (median, IQR)	29 (10–49)	26 (11–52)	42 (11–65)	46 (1–86)
Pain (median VAS, IQR)	24.5 (10.75–46.25)	27 (10–49)	36 (7–66)	47 (2–95)
HAQ score (median, IQR)	0.5 (0.13–1.13)	0.25 (0–1)	0.4 (0.1–1.28)	0.89 (0–2.75)
DAS28 (median, IQR)	3.13 (2.03–4.65)	n.d.	n.d.	3.6 (0.53–7.57)

*RA, rheumatoid arthritis; n, number; IQR, interquartile range; MTX, methotrexate; DMARD, disease-modifying antirheumatic drug; CCP, citrullinated protein; RF, rheumatoid factor; seropositive is defined as RF positive, CCR positive or both; seronegative is defined as RF and CCP negative; CRP, C-reative protein, VAS, visual analog scale; HAQ, health assessment questionnaire; DAS28, disease activity score 28; n.d, no data*.

* *% of the total patients within the cohort*.

In the prospective study, all RA patients initiated on infliximab from the Sahlgrenska University Hospital between 2017 and 2019 were included. Patients in the prospective cohort (*n* = 73) were included prior to initiation of infliximab treatment and followed for up to 1 year. All patients but one (previously treated with infliximab 2011-2012 and golimumab December 2016 to December 2017), were naïve to infliximab treatment at baseline. The majority of patients were concurrently treated with methotrexate, either alone (*n* = 52) or in a combination with salazopyrin (*n* = 5), plaquenil (*n* = 2) or prednisolone (*n* = 9) at the initiation of infliximab therapy. Four patients received concomitant salazopyrin only and one patient was treated with infliximab monotherapy. The patients treated at Sahlgrenska received a dosing schedule as follows; baseline, the second dose was received after 2 weeks, the third dose after 1 month, and thereafter, every 8 weeks. For this cohorts, serum samples were collected at baseline and trough prior to each infusion. The infliximab dosing regimen for this cohort was 200 mg intravenous infusion administered every 8 weeks. Patients that failed to respond were given either an increased dose and/or shortened treatment intervals or were switched to another treatment. This decision was made by the treating physician.

All patients signed informed consent to participate in this study, which was approved by Stockholm Regional Ethical Committee (2013/1034-31/3) and Gothenburg Regional Ethical Committee (1028-15, 2016-02-12).

### Measurement of Clinical Data

Routine clinical examinations of patients were performed by treating rheumatologists at regular intervals according to local clinical practice, the Disease Activity Score in 28 joints (DAS28) was calculated and data registered in the Swedish Rheumatology Quality Register (SRQ). The first visit to evaluate the treatment response usually occurred 3–4 months after initiation of infliximab treatment. Clinical data including disease duration, rheumatoid factor (RF)/cyclic citrullinated peptide (CCP) status, smoking habits, and concomitant csDMARD treatment was retrieved from SRQ and patients' medical records. Seropositivity was defined as being CCP and/or RF positive and seronegativity was defined as being CCP and RF negative.

### Assessment of Disease Activity

The composite disease activity score (DAS28) was used. The DAS28 score takes into account the number of swollen and tender in a 28-joint count, the erythrocyte sedimentation rate (ESR) or C-reactive protein (CRP) and patient's assessment of global health on a visual analog scale (VAS-GH). Based on this score, patients were classified into the following categories: remission DAS28 <2.6, low disease activity DAS28 2.6–3.2, moderate disease activity 3.2–5.1, and high disease activity >5.1. The change in a patient's score over time, is expressed as delta (Δ) DAS28, was calculated by subtracting baseline DAS28 score from the respective final post-treatment score. Using the ΔDAS28, patients were categorized as either good, moderate, or non-responders to infliximab treatment according to the European League Against Rheumatism (EULAR) response criteria as previously described (30). In short, good responders are defined as those with a ΔDAS28 >1.2 and a current DAS28 ≤ 3.2; moderate responders by a ΔDAS28 >1.2 and a current DAS28 >3.2 or a ΔDAS28 between 0.6–1.2 and a current DAS28 ≤ 5.1; non-responders were defined as those with a ΔDAS28 <0.6 or ΔDAS28 between 0.6–1.2 and a current DAS28 score >5.1. For the cross-sectional cohort, the DAS28 used for analyses were assessed within 3 months from the infliximab trough drug level measurement which was used for the study analyses.

### ELISA for Detection of Infliximab Serum Trough Levels

Infliximab trough level was measured in patient sera using an in-house developed and validated ELISA which is used in clinical routine, as previously described (28). Briefly, microtiter plates (Nunc Maxisorp F 96, Thermo-Fisher Scientific, Roskilde, Denmark) were coated with 50 μL per well of recombinant human TNF-α (200 ng/mL) (R&D Systems, Minneapolis, MN, USA) in 0.05 M sodium carbonate buffer pH 9.6. The plates were put on a shaker at room temperature (RT) for 2 h before being incubation overnight in +4°C. The plates were then washed three times with phosphate buffered saline (PBS) plus 0.05% pH Tween 20 and blocked with PBS + 1% bovine serum albumin (BSA) (Sigma, St. Louis, MO, USA) and 0.05% Tween 20 (blocking buffer) for 1 h at RT. After washing, standard dilutions (0.40–100 ng/mL) of infliximab (Schering Plough, Kenilworth, NJ, USA), internal controls (defined IFX-spiked sera), and patient samples, diluted 1/500 in blocking buffer, in duplicates were added to the plate. Plates were incubated on a shaker at RT for 1 h and washed four times before addition of alkaline phosphatase (ALP)-conjugated goat anti-human IgG (Fc-specific) (Sigma) diluted 1/10 000 in a blocking buffer. The plates were again incubated on a shaker at RT for 1 h and washed four times. The substrate (p-nitrophenyl-phosphate, 5 mg/mL in 1 M diethanolamine with 0.5 mM Mg, pH 9.8) was added and color development was monitored at 405 nm. The concentration of samples and controls was calculated from the standard curve where the lower and upper limits of quantification were 0.2 and 50 μg/mL, respectively (compensated for serum dilution 1/500).

### Inhibition ELISA for Detection of Antibodies to Infliximab

ADA to infliximab was detected using an in-house developed and validated ELISA, which is based on the inhibition of labeled infliximab binding to TNF-coated ELISA plates, as previously described (28). Alkaline phosphatase (ALP) was coupled to infliximab using the Lightning-Link kit (Innova Biosciences Ltd., Cambridge, UK). The ELISA plates were coated with TNF-α, as described for the infliximab ELISA above. A standard consisting of goat anti-human IgG (Jackson Immuno Research) at a final concentration of 1 μg/mL was used. Patient sera were analyzed at final dilutions of 1:10 and 1:100. The standard defined control sera and patient samples were incubated with ALP-conjugated infliximab for 1 h at RT. After an additional wash of the TNF-coated plate, aliquots of standard, controls and patient samples were transferred to the plate in duplicates. After incubation on a shaker for 1 h at RT, the plate was washed and substrate (p-nitrophenyl-phosphate, 5 mg/mL in 1 M diethanolamine with 0.5 mM Mg, pH 9.8) was added, and color development was monitored at 405 nm. The results were transformed to percentage inhibition by normalization of the OD of the samples to that of the standard (100% inhibition) using the formula (OD blank – OD sample)/(OD blank – OD standard) x 100. The lower limit of detection was set to the value plus two standard deviations obtained from measurements of normal control sera. Due to free infliximab interference with the assay, ADA could only be detected in the absence of the drug. ADA analysis was therefore limited to patient samples where serum infliximab was undetectable (<0.2 μg/mL).

### ADA Detection With the Precipitation and Acid Dissociation (PandA) Method

Presence of ADA to TNF-α inhibitors was assessed using the PandA method described by Zoghbi et al. (27) on the Meso Scale Discovery® (MSD) platform. The PandA assay has demonstrated high sensitivity to detect ADA in the presence of a high concentration of drug (drug tolerant). The assay is therefore, more suitable for immunogenicity assessment of patient samples that contain detectable levels of infliximab which interfere with the detection limit of ADA in other immunoassays. Serum from 40 healthy donors (Stockholm blood center, Sweden) was collected to prepare a pool of normal healthy sera (NHS) to be used as negative control (NC) in the PandA assay. Informed written consent was given by all the donors. Human anti-IFX (clone HCA233, BIO-RAD) was prepared in NHS to be used as a high positive control (6 μg/mL) and low positive control (1 μg/mL). The PandA assay was validated in-house before analysis of study samples. A plate specific, or floating, cut-point was applied to allow a 5% false positive rate in the screening assay. For each plate, the cut-point is calculated by multiplying the NC value of the plate with a normalization factor (NF), which is the average of all NC values obtained from six assay plates in the validation process (three operators running one plate each over two days). To resolve the problem of interference of soluble infliximab in the sera, 25 μL of patient samples were diluted 1:2 in 2% bovine serum albumin (BSA, Sigma) in PBS containing 10 μg/mL infliximab (Remicade®) in a polypropylene V-bottom plate (Thermo Scientific) in duplicate wells. ADA present in the sera were then able to bind to the excess soluble infliximab, forming immune complexes during a 1 h incubation at RT at 450 rpm. Formed immune complexes were then precipitated by addition of 50 μL 6% polyethylene glycol (PEG, Aldrich) solution to each sample (3% PEG in the plate) during overnight incubation of the plate at 4°C. The following day, the plate was centrifuged at 3,724 x g for 30 min to precipitate immune complexes into a pellet. After the supernatant was discarded, the pellets were re-suspended with a 3% PEG solution and the plate centrifuged at 3,724 g for 20 min. This step was repeated once more. After the final centrifugation step, pellets were re-suspended with 250 μL 0.25 M acetic acid, pH 3.0, to get a minimal required dilution of 1/10. Samples were thereafter transferred to a high binding carbon plate (MSD) by adding 50 μL of each sample in duplicates. The plate was thereafter incubated for 1 h at RT at 450 rpm. Following incubation, the plate was washed once with 300 μL 1x wash buffer (1xPBS, 0.1% Tween, pH 7.4) and blocked with 300 μL casein in PBS pH 7.4 (Thermo Scientific) for 1 h at RT at 450 rpm. After additional washing, 50 μL of the master mix containing 0.5 μg/mL of Sulfo-Tag conjugated infliximab (labeling ratio of 20:1 between Sulfo-Tag and IFX) (MSD GOLD^TM^) in 2% BSA in PBS was added to the samples and incubated for 1 h at RT at 450 rpm. After the final incubation, the plate was washed once and within 5 min of adding 150 μL read buffer T (2x) (MSD) the plate was read on MESO Quickplex SQ 120 (MSD). By electrical discharge, the electrons are excited and a stable light signal is generated. This electrochemiluminescence (ECL) signal is proportional to the amount of ADA in each serum sample. A signal to background ratio was calculated by dividing the average ECL signal from an individual sample by the average ECL signal of the negative control (NHS) and expressed as relative ECL (RECL). The coefficient of variation value of ≤25% was accepted as the maximum variation between duplicates.

### Neutralizing Anti-drug Antibody Analysis

The neutralizing capacity of the ADA were measured using iLite^TM^ infliximab NAb bioassay (Biomonitor) in 35 serum samples from 29 patients in the cross-sectional study, who previously detected ADA-positive by the ELISA method. The protocol was carried out according to the manufacturers' instructions. In short, the assay uses division-arrested TNF-α sensitive cells to measure TNF-α bioactivity. Transcription of the luciferase gene occurs when TNF-α binds to the TNF-α receptor and the luciferase activity is inversely proportional to the amount of infliximab present in the sample. Luciferase activity was measured using GloMax Luminometer (Promega) and the antibody neutralizing activity was normalized to Renilla (31). The assay's drug tolerance is 0.65 μg/mL.

### Statistical Analysis

To compare continuous variables, Mann-Whitney *U*-test for independent groups was used. All reported *p*-values were two tailed, and a *p-*value of < 0.05 was considered statistically significant. Correlation analysis was performed using Spearman's rank correlation. Receiver operating characteristic (ROC) curves were constructed to assess the cut-off of drug level- and ADA threshold values were based on EULAR response criteria (good responders vs. non-responders). The cut-off points were calculated on the basis of the best trade-off values between sensitivity and specificity. Statistical calculations were performed using Prism software (GraphPad Inc. version 8).

## Results

### Patient Characteristics

Baseline patient characteristics of the infliximab-treated cohorts are summarized in Table 1. Patients in the prospective cohort had a median disease duration of 1.5 years (IQR 0.5–11) at baseline. In contrast, patients in the cross-sectional cohort had a wide-ranging disease and treatment duration at the time of baseline sampling. The study time points of sample collection for the prospective cohort and diagnoses for the cross-sectional cohort are illustrated in Figures S1, S2.

### Serum Infliximab Levels

**Figure 1:**
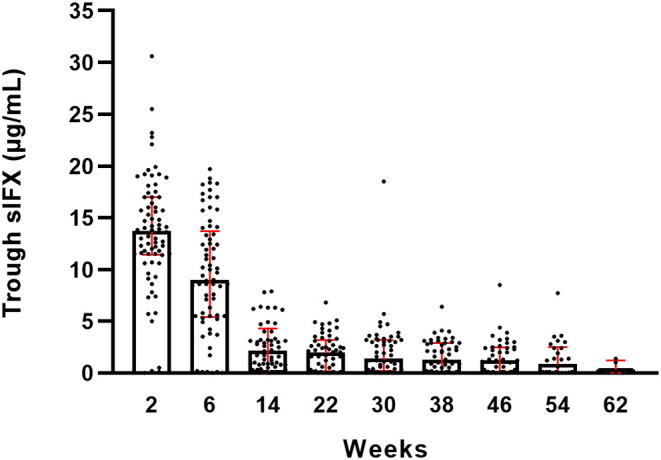
Trough serum infliximab levels (sIFX) for all RA patients in the prospective cohort and variation over time. Total number of weeks on treatment is shown on the x-axis. The levels are presented as median (bar) and interquartile range (red). Number of individuals at each time point is illustrated in Figure S1A.

**Figure 2:**
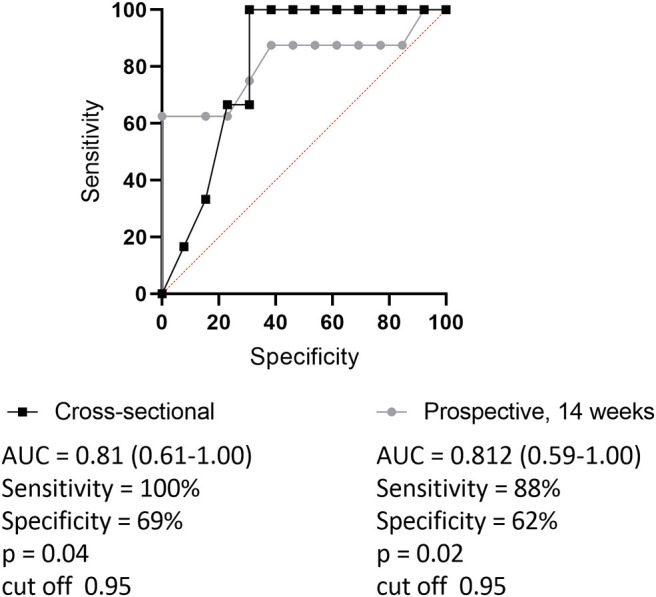
Receiver operating characteristic curve (ROC) for setting optimal cut-point of trough sIFX separating good from non-responders (based on EULAR criteria). Black line with quadrates shows cross-sectional cohort and gray dotted line represents prospective cohort. The levels of trough sIFX of the prospective cohort were taken at 14 weeks after treatment initiation. Area under the ROC curve (AUC) was 0.81 and the cut off level was 0.95 for both cohorts.

The sIFX values in the prospective study were found to be higher at the beginning of the treatment period when the intervals were 2 and 4 weeks between infusions. It then reached a stable range with a mean of 1.8 μg/mL [standard deviation (SD) of 2.0 μg/mL] from week 14 when infusions were 8 weeks apart (Figure 1). To determine an optimal sIFX level defined as the range of trough levels that had the sensitivity and specificity to predict good EULAR response, Receiver Operating Characteristic (ROC) curves where done for both the cross-sectional cohort and the prospective cohort (Figure 2). On the analysis, the therapeutic ranges were distributed in a U-shaped curve at the lower and upper ends. Therefore, each end were analyzed separately. Firstly, the lower end was determined on all samples with a sIFX under 3.8 μg/mL, corresponding to the mean + SD for the stable range. With an area under the curve (AUC) of 0.8, both cohorts gave a predictive optimal value of 0.95 μg/mL indicating this is the lowest sIFX level which you can expect the treatment to have good effect. For ROC analysis, the EULAR response was used as the clinical outcome and for the prospective cohort both EULAR response and remission status were used.

Using the previously suggested lower limit for optimal therapeutic effect of 1 μg/mL sIFX (32) and the data from the ROC analyses (taking into account the interassay variations), an approximate lower end of an optimal range was set to 1 μg/mL. Secondly, an upper end for the optimal range was estimated using the mean + 2xSD of the stable sIFX period (from week 14 onward) in the prospective cohort, which was found to be 6 μg/mL sIFX in trough. This is in line with what has been shown previously for other TNFi (28, 33).

**Figure 3:**
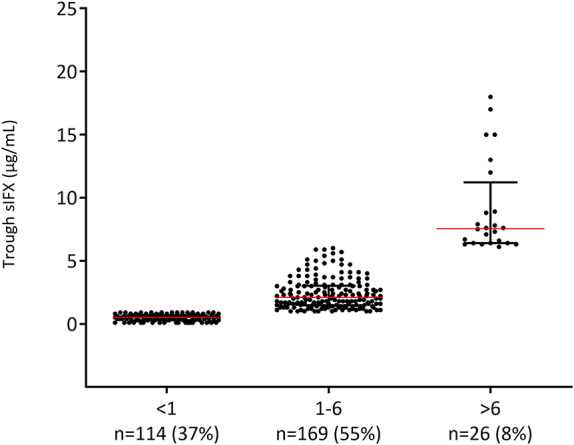
Levels of trough serum infliximab (sIFX) in RA patients in the cross-sectional cohort. Samples divided based on suggested optimal levels (1–6 μg/mL) of trough sIFX. The levels are presented as median (red line) and interquartile range (whiskers).

Using this range (1–6 μg/mL) as optimal, only 55% of the samples (*n* = 169) from RA patients in the cross-sectional cohort had an optimal sIFX, 37% had lower than 1 μg/mL, and 8% had higher than 6 μg/mL (Figure 3). The proportions were similar for the other non-RA infliximab treated patients in this cohort, where 65% of the samples (*n* = 273) had an optimal sIFX, 24% had lower than 1 μg/mL, and 11% had sIFX higher than 6 μg/mL.

In the prospective study, only 54% of all samples (*n* = 159) collected from 65 patients from week 14 onwards had sIFX levels within the suggested optimal range, 42% of the samples had sIFX levels <1 μg/mL and 4% had a level higher than 6 μg/mL.

As a comparison, five samples which had been mistakenly collected directly after the infusion, were found to have a mean sIFX level of 46 μg/mL (median 48 μg/mL, range 42–52 μg/mL). These were not included in the analyses.

### Clinical Relevance of Serum Infliximab Levels

**Figure 4:**
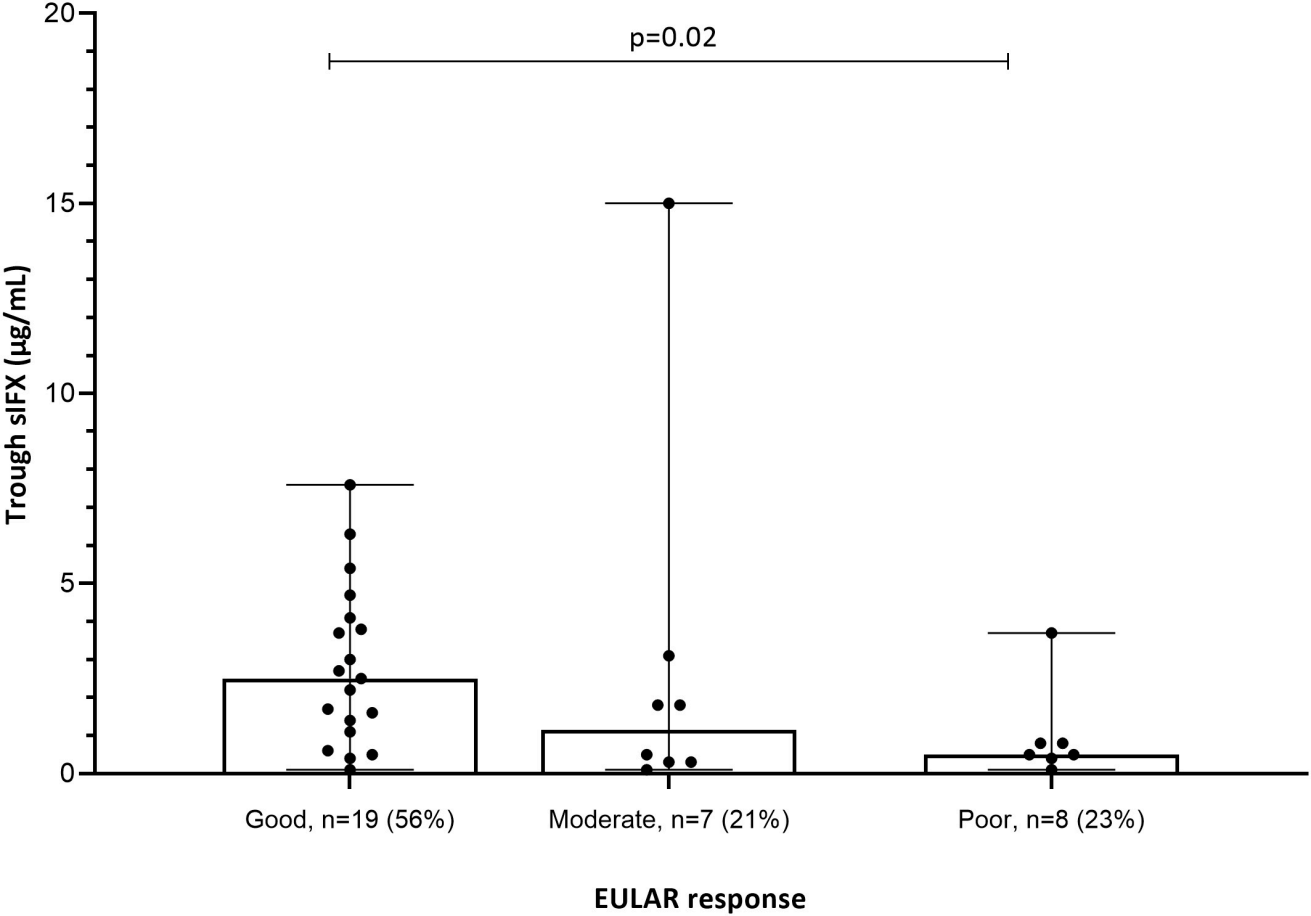
EULAR response in relation to sIFX levels for the subgroup of RA patients in the cross-sectional cohort (*n* = 34). The sIFX levels were significantly lower in the non-responders group compared to the good responders. Percentage of patients in each EULAR group given in brackets on x axis. The levels are presented as median (bar) and interquartile range (whiskers).

**Figure 5:**
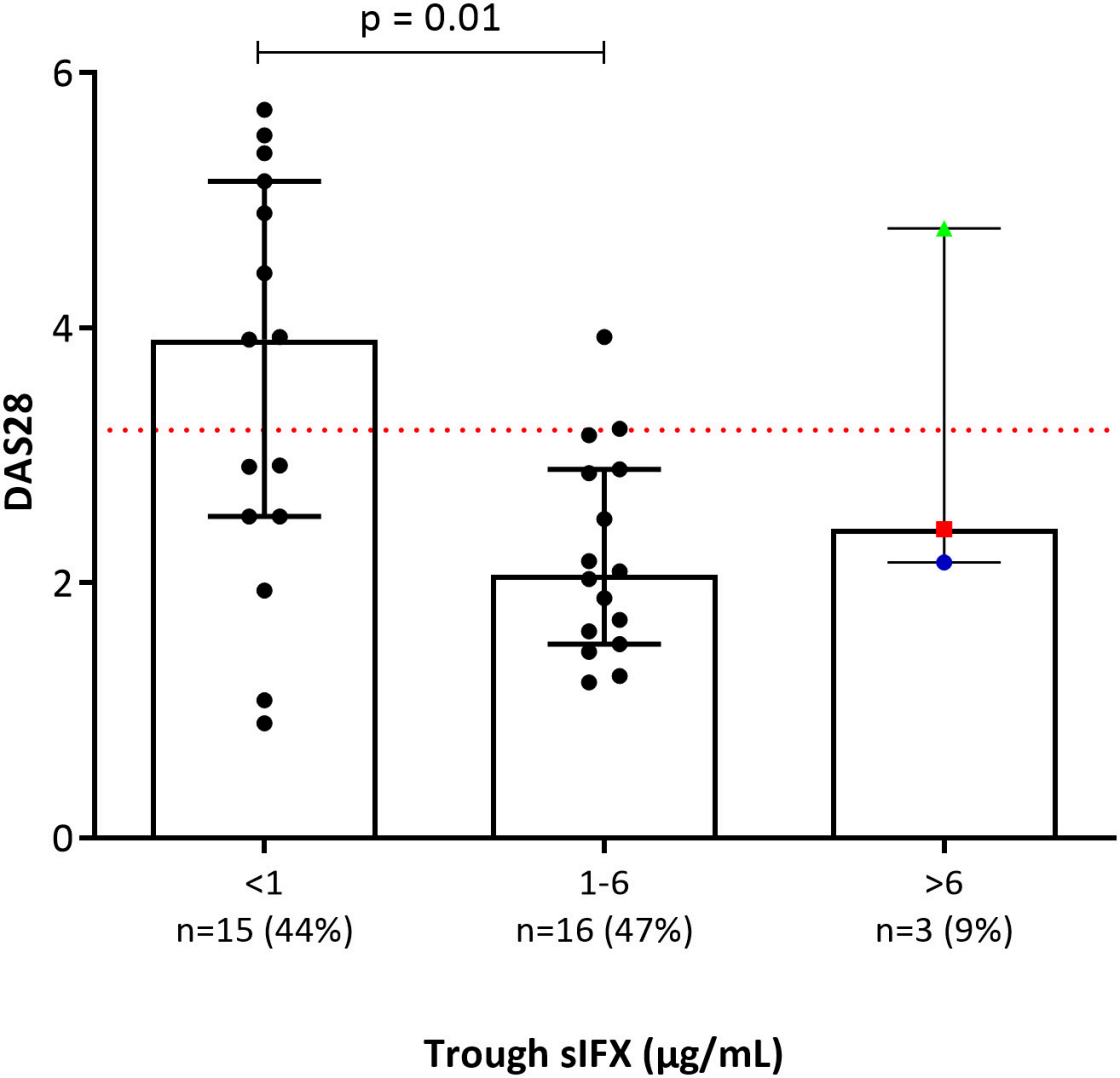
Bar plot of DAS28 levels in a subgroup of the RA patients in the cross-sectional cohort (*n* = 34). Patients with RA were divided into three groups based on trough sIFX: under 1, 1–6, and above 6 μg/mL. DAS28 was significantly worse in patients with a sIFX trough level below 1 μg/mL compared with those with a drug level between 1 and 6 μg/mL. Dotted line depicts DAS28 of 3.2 and indicates low disease activity bellow this line. The trough sIFX levels presented as median (bars) and interquartile range (whiskers). Colored dots represent 3 RA patients who had sIFX >6 μg/mL.

**Figure 6:**
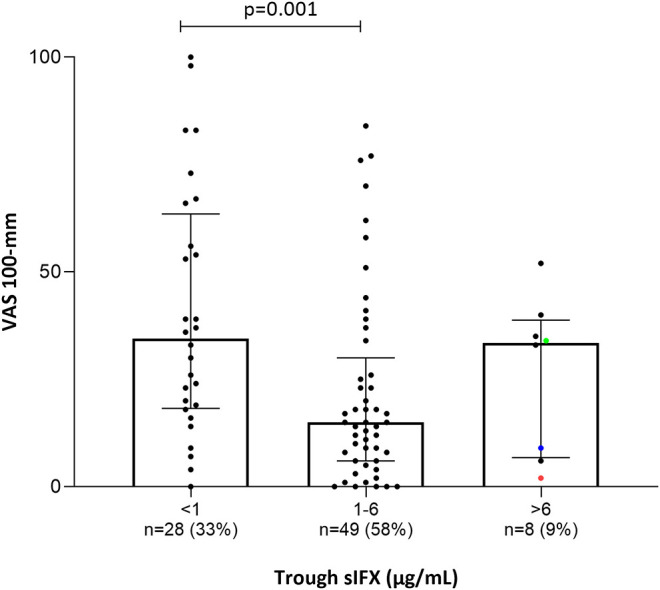
Bar plot of the levels of patients' pain assessment (visual analog scale 100 mm; VAS) in all patients (with available data) treated with infliximab in the cross-sectional cohort. Patients were divided into three groups based on trough sIFX: under 1, 1–6, and above 6 μg/mL. VAS was significantly higher in patients with a sIFX trough level below 1 μg/mL compared with those with a drug level between 1 and 6 μg/mL. The trough sIFX levels presented as median (bars) and interquartile range (whiskers). Colored dots represent 3 RA patients.

**Figure 7:**
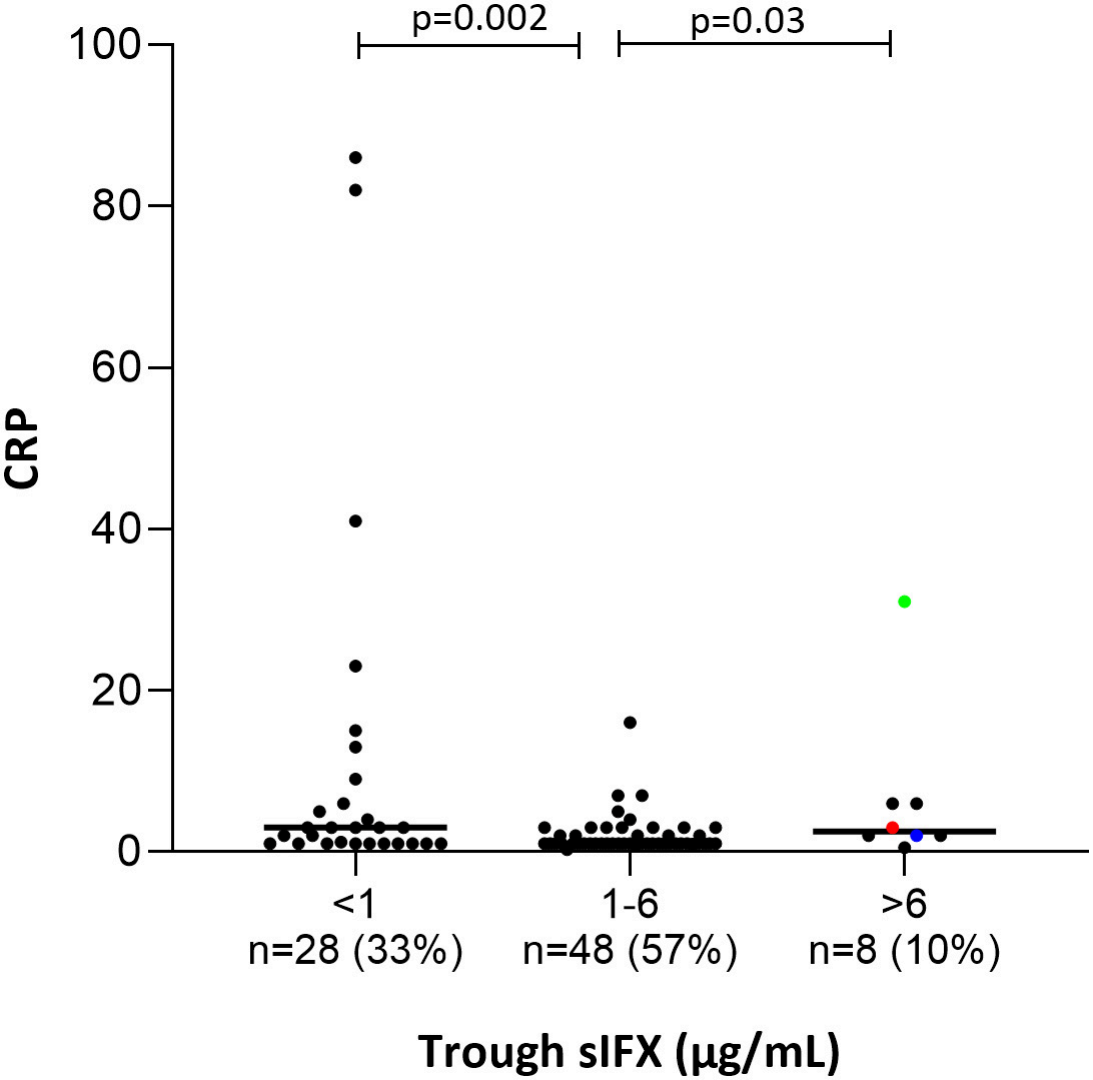
Bar plot of the levels of C-Reactive Protein (CRP) in all patients (with available data) treated with infliximab in the cross-sectional cohort. Patients were divided into three groups based on trough sIFX: under 1, 1–6, and above 6 μg/mL. CRP was significantly higher in patients with a sIFX trough level below 1 and above 6 μg/mL compared with those with an optimal drug level. The trough sIFX levels presented as median (bars) and interquartile range (whiskers). Colored dots represent 3 RA patients.

**Figure 8:**
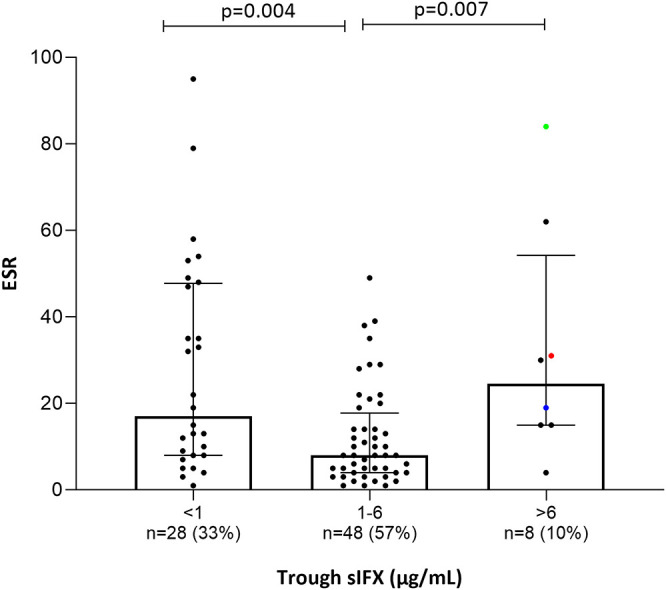
Bar plot of the levels erythrocyte sedimentation rate (ESR) in all patients (with available data) treated with infliximab in the cross-sectional cohort. Patients were divided into three groups based on trough sIFX: under 1, 1–6, and above 6 μg/mL. ESR was significantly higher in patients with a suboptimal sIFX trough level (below 1 and above 6 μg/mL) compared with those with a drug level between 1 and 6 μg/mL. The trough sIFX levels presented as median (bars) and interquartile range (whiskers). Colored dots represent 3 RA patients.

Clinical variables were used to analyse the correlation between sIFX and treatment response in the cross-sectional cohort. For RA patients in the cross-sectional cohort, if a DAS28 score was available within 5 months of the time of sampling (*n* = 34) this was also used to evaluate the correlation. Using the DAS28, patients were then classified as previously described using the EULAR response with good, moderate or non-response categories (30). The sIFX levels were significantly lower in the non-responders group compared to the good responders (Figure 4). In the moderate responder group, there is one outlier case with a high sIFX level (15 μg/mL), indicating that also a high drug level might be associated with less optimal therapeutic response. The cross-sectional cohorts' sIFX levels were divided into the categories of under 1, 1–6, and above 6 μg/mL and analyzed for correlation with clinical variables, including DAS28 (*n* = 34) (Figure 5) and delta-DAS28 (*n* = 34) (Figure S3) for the RA patients. Variables on patient global assessment (PGA) (Figure S4), patient pain assessment (VAS) (Figure 6), CRP (Figure 7), and ESR (Figure 8), were analyzed for all infliximab treated patients in the cross-sectional cohort. Clinical variables were significantly worse in patients with a sIFX trough level below 1 μg/mL compared with those with a drug level between 1 and 6 μg/mL (DAS28 (*p* = 0.01), PGA (*p* = 0.01), CRP (*p* = 0.002), and ESR (*p* = 0.004).

Only a few patients had sIFX >6 μg/mL (*n* = 8, of which 3 were RA patients) and both CRP (*p* = 0.03) and ESR (*p* = 0.007) were significantly worse in these compared with those with a drug level between 1 and 6 μg/mL. One possible reason for a very high sIFX level could be the presence of more severe disease at treatment initiation and therefore, dosing intervals are shortened or doses increased in attempt to manage the patients' symptoms. Patients from the cross-sectional cohort with sIFX >6 μg/mL did indeed have both a higher DAS28 and a higher treatment dose (Figure S5).

In the prospective cohort the correlation between EULAR response and sIFX levels varied between time points, but collectively, the non-responders had a significantly lower sIFX level than the good responders (Figure S6).

### Proportion of Patients With Free ADA

Given that the drug sensitive ELISA method only reliably detects free ADA, not bound in immune complexes, it is recommended that only samples with sIFX below the drug sensitivity of the assay (<0.2 μg/mL) are tested. Of the 73 patients in the prospective cohort, 44 (60%) had a sIFX <0.2 μg/mL at some time point allowing the sample to be tested for ADA with ELISA. Of these 44, 86% (*n* = 38), or 59% of the whole cohort, were positive for ADA. Compared to this early RA cohort, a lower proportion of the cross-sectional cohort, or 14% (37 of 270 patients) of the whole cohort were found to be ADA positive in samples with sIFX levels <0.2 μg/mL (42 of 270), probably reflecting a selection bias of patients who continue on infliximab.

### ADA in Samples With Detectable sIFX Level Using PandA

**Figure 9:**
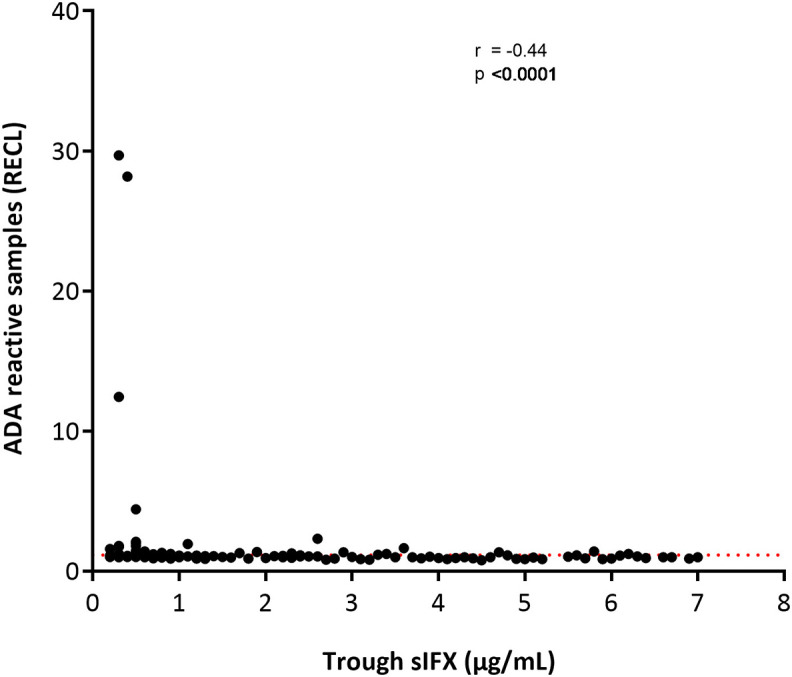
Correlation between ADA reactivity (in relative ECL; RECL) and trough sIFX levels in cross-sectional cohort. A significant reverse correlation was found between ADA and sIFX levels with *r*^2^ of −0.4 and *p* < 0.0001. ADA reactivity was detected with PandA assay. Dotted line separates samples positive for ADA.

**Figure 10:**
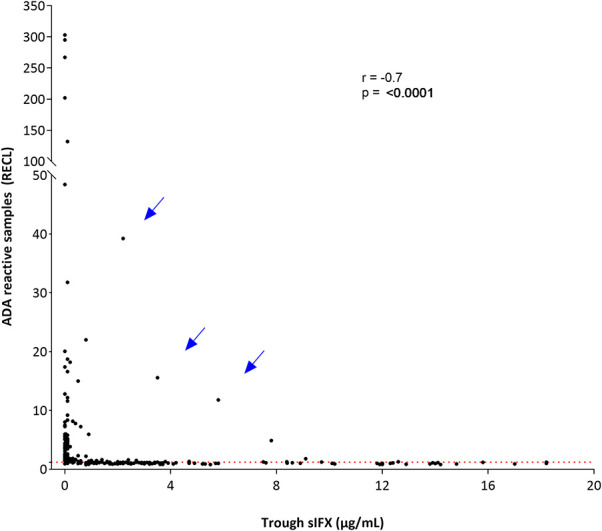
Correlation between ADA reactivity and trough sIFX levels in prospective cohort. A significant reverse correlation was found between ADA and sIFX levels with *r*^2^ of −0.7 and *p* < 0.0001. ADA reactivity was detected with PandA assay. Dotted line separates samples positive for ADA. Arrows pointed out at three exceptional cases with a high levels of trough sIFX and ADA simultaneously.

With the drug-tolerant PandA method, ADA can be detected regardless of drug level, and therefore we could determine the frequency of ADA in samples with sIFX >0.2 μg/mL. A selection of samples from the cross-sectional cohort with sIFX levels ranging 0.2–7 μg/mL were analyzed with PandA (Figure 9). Only a 26 additional ADA positive samples were identified with this method and for the cross-sectional cohort all had a sIFX levels under 1 μg/mL. Similar results were found in the prospective cohort, with three interesting exceptions described more in detail below (Figure 10). A significant reverse correlation was found between ADA and sIFX levels in both cohorts (*r*^2^ = −0.4, *p* < 0.0001 for cross-sectional cohort and *r*^2^ = −0.7, *p* < 0.0001 for the prospective cohort).

In the prospective cohort, the kinetics of total (free and IC bound) ADA development was determined using the drug-tolerant PandA assay. ADA was found to develop early after treatment initiation and the first positive samples could already be identified prior to the 2nd infusion at week 2. The incidence of ADA increased until the 6th infusion at week 30, but cases with their first positive sample were detected up until the 9th infusion at week 54. The cumulative prevalence of ADA increased up to the 6th infusion at week 30 before beginning to decrease (Figure S7).

### ADA Results From ELISA Compared to PandA

Of the 108 selected prospective samples tested on both PandA and ELISA methods, there was a 72% (*n* = 78) agreement between assay results [68% (*n* = 74) confirmed positive and 4% (*n* = 4) confirmed negative]. However, there were some discrepancies between assays, with 20% (*n* = 22) positive in PandA but negative with ELISA, and 7% (*n* = 8) positive with ELISA but negative with PandA.

Of the five patients with sIFX levels <0.2 μg/mL that were negative for ADA with ELISA, one was confirmed positive with PandA.

### ADA in Complex With Drug Can Be Detected Earlier Than Free ADA

In the prospective cohort, 45 patients from whom we could retrieve samples which were taken prior of being identified as ADA positive with ELISA, were tested for ADA with PandA. Of these, 17 patients (38%) were found to be ADA positive at an earlier time point. These were on average detected 20 weeks (median 16, range 4–41 weeks) before the sIFX levels were low enough for detection of ADA with ELISA.

Three unique cases were identified which were found to be ADA positive despite very high sIFX and therefore serial samples from these cases were analyzed (Table 2). The first case was newly diagnosed and was trialled on methotrexate for 2 months without effect before initiating the first infliximab infusion. This patient was highly ADA positive (PandA RECL = 10) already at the 2nd infusion, just 2 weeks after treatment initiation. As the sIFX was 5.8 μg/mL, this patient would not have been tested for ADA with ELISA until the next infusion at week 6, when the drug level was <0.2 μg/mL. In this sample the ELISA test could then confirm ADA positivity. An infusion reaction was noted at the third infusion, which worsened despite precautions taken before the 4th infusion. The patient discontinued treatment after the 4th infusion due to infusion reactions and lack of treatment effect. The patient was switched to rituximab treatment.

**Table 2:** Three unique cases which were found to be ADA positive despite very high sIFX.

	**Infusion of Infliximab**							
	**2nd**	**3rd**	**4th**	**5th**	**6th**	**7th**	**8th**	**9th**
**Case #1**								
**PandA, RECL**	**10**	**16**	**31**	n/a	n/a	n/a	n/a	n/a
**sIFX**	5.8	<0.2	<0.2	n/a	n/a	n/a	n/a	n/a
**ELISA**	n/a	pos	pos	n/a	n/a	n/a	n/a	n/a
**Case #2**								
**PandA, RECL**	**75**	**48**	**39**	**39**	**22**	**18**	**12**	**12**
**sIFX**	12.2	8.6	2.6	2.2	0.8	0.2	0.1	0.1
**ELISA**	n/a	n/a	n/a	n/a	neg	**pos**	**pos**	**pos**
**Case #3**								
**PandA, RECL**	1	**15**	**5**	**23**	**267**	**355**	**394**	n/a
**sIFX**	15.7	3.5	0.05	0.0	0.0	0.0	0.0	n/a
**ELISA**	n/a	n/a	**pos**	**pos**	**pos**	**pos**	**pos**	n/a

*Serial samples from these cases were analyzed. ADA was measured by Elisa and PandA assays. In all of these cases ADA have been detected with PandA already at the 2nd or 3rd infusion. Bold indicates positive ADA values*.

The second case was also newly diagnosed and was treated with prednisolone and methotrexate for 6 months prior to their first infliximab infusion. This patient was also highly ADA positive (PandA RECL = 75) already at the 2nd infusion (week 2) and with a sIFX level of 12.2 μg/mL. This patient was tested for ADA with ELISA in a subsequent sample taken at the 6th infusion, 28 weeks after treatment initiation with a serum infliximab of 0.8 μg/mL. At this point, the patient was found to be ADA negative with ELISA and still positive with PandA (RECL = 22). At the 7th infusion, the sIFX level had decreased to 0.2μg/mL and at this time point, the patient was first detected as ADA positive using the ELISA. This was 37 weeks after the first ADA positive detected using the PandA method. At this time, the patient experienced good effect of the treatment with a DAS28 of 2.8. A year later the treatment effect declined and the interval was shortened to 6 weeks without effect. The patient was then switched to etanercept.

The third case was newly diagnosed with low disease activity (DAS28 at treatment initiation 3.02) and was initially managed with intra-articular cortisone injections. Methotrexate was initially used but terminated due to a herpes zoster infection, which was managed with vaccination and anti-viral therapy. Infliximab treatment was started 2 years later, initially with good effect. This patient was ADA negative in PandA (RECL = 1) prior to the 2nd infusion, but highly positive prior to the 3rd infusion (RECL = 15) when the sIFX was still high (3.5 μg/mL). In the sample taken prior to the 4th infusion, sIFX was 0.1 μg/mL and ADA positive with ELISA, and PandA however, the PandA positive level had dropped to 5 RECL. With the subsequent infusions the ADA increased to very high levels (RECL = 394 prior to the 8th infusion). An infusion reaction was documented after the 5th infusion and fevers after infusion 6 and 7, followed by facial skin reactions after infusion 8. The infusion reaction worsened after the 9th infusion and the patient was then shifted to adalimumab.

### Clinical Threshold Value for ADA

When ADA positivity measured by PandA was stratified by patients' EULAR response categories good, moderate or non-responder, a notable difference between the proportion of ADA positive patients was observed between groups (Figure S8). However, this was less clear over time (Figure S9). Overall, a higher mean ADA value was found in the group with EULAR non-responders (15.7 RECL), whereas 79% of patients in the moderate and good EULAR response groups had a ADA result of <3 RECL. This suggests that there is a clinical threshold value for ADA of around 3 RECL, using this method. A ROC analysis using the RECL values from prior to the 4th infusion (week 14) and the overall EULAR outcome (good and non-responders) of the patients in the prospective study, confirmed a clinical threshold value of 3.25 (AUC 0.9, *p* = 0.028) (Figure S10). When remission outcomes were also used, the clinical threshold value was 1.48 (AUC 1.0, *p* = 0.01) (Figure S10).

### ADA Correlation to Seropositivity and to Smoking Status in RA Patients

**Figure 11:**
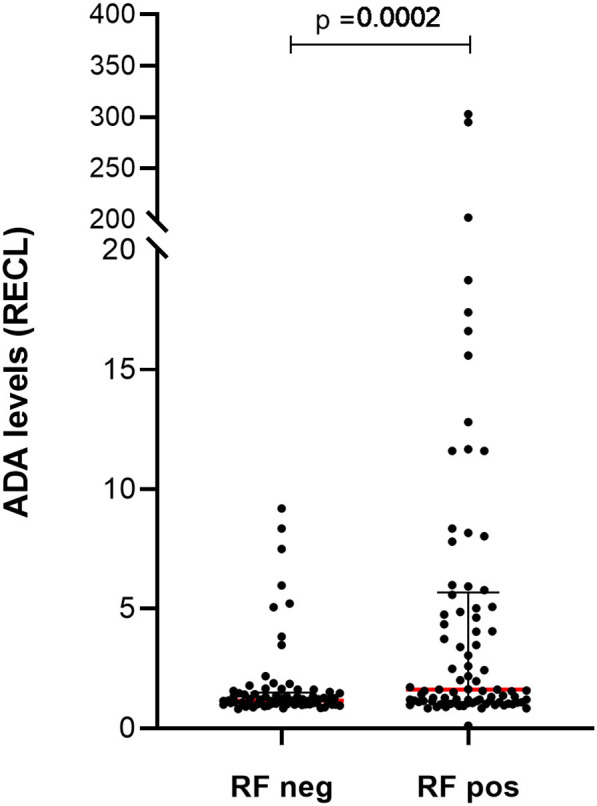
Levels of ADA vary between patients with and without rheumatoid factor (RF) in prospective cohort. Significantly higher levels of ADA were detected in samples (*n* = 89) from patients with RF compared to the samples (*n* = 73) from those patients who were RF negative. The ADA levels presented as median (red lines) and interquartile range (whiskers).

In the prospective cohort, the level of ADA, as measured with PandA method, had a positive correlation with serological status of the patients (Figure S11). Over time, the most prominent correlation was noted prior to infusion 8 (Figure S12). This effect was due to the RF and when analyzed separately, patients who were RF positive had significantly higher ADA RECL values (Figure 11), seen also over time (Figure S13).

**Figure 12:**
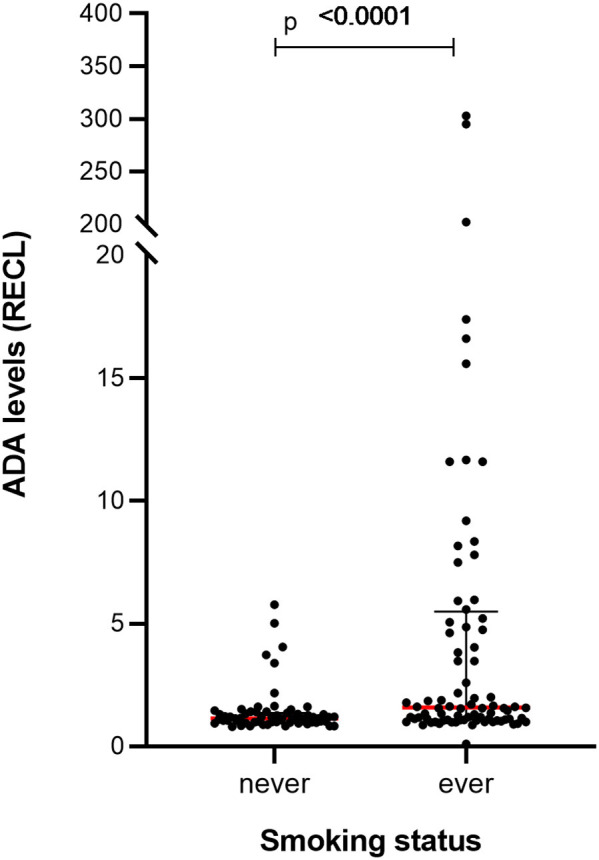
Levels of ADA vary between RA patients with a different smoking status in prospective cohort. Significantly higher levels of ADA were detected in the samples (*n* = 84) from ever smokers compared to the samples (*n* = 63) from patients who had never smoked. The ADA levels presented as median (red lines) and interquartile range (whiskers).

The smoking status of the patients was available for 54% of patients, and a highly significant correlation in ADA levels was seen between ever smokers (*n* = 84) and never smokers (*n* = 63) (*p*-value = 0.0001) (Figure 12), a pattern that could also be noted over time (Figure S14).

### ADA to Infliximab Had Neutralizing Capacity

In the cross-sectional cohort, serum samples from 29 patients who tested positive for free ADA with the ELISA, were further tested for neutralizing capacity using the iLite bioassay. Nineteen of those patients (66%) tested positive for neutralizing ADA against infliximab. There was a strong correlation between assays (*r* = 0.9, *p* < 0.001), particularly in those with RECL values above the clinical threshold value (RECL > 3) (Figure S15).

### Transient and Persistent ADA

In the prospective cohort, 39 patients were identified as ADA positive with PandA method, and 29 of these had one or more samples taken after they first were determined to be positive. Therefore, they could be assigned as transient or persistent positive. The majority of these were persistent positive (*n* = 22, 56%) and had a higher peak RECL (median of 5.1, IQR 2.2–14.4) compared to the transient positive (*n* = 7, 18%) with a peak RECL value of 1.2 (IQR 1.2–1.3). Notably these groups were on either side of the suggested clinical threshold value for ADA (1.48–3.26).

### Therapeutic Drug Monitoring Test Algorithm

**Figure 13:**
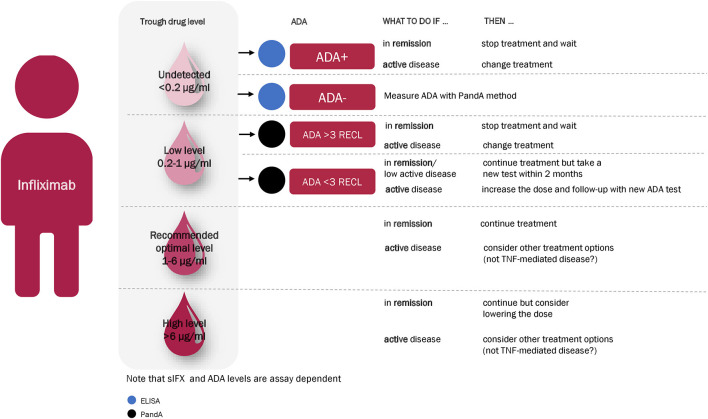
Recommended infliximab treatment algorithm for RA based on the results of this study. With an algorithm starting with a measuring of the sIFX level at 14 weeks after treatment initiation, patients divided into four main categories. **Group 1** and **Group 2** consists of patients with either undetectable infliximab levels (<0.2 μg/mL) or low sub-therapeutic sIFX trough levels (<1 μg/mL) and should therefore be tested for ADA with ELISA or a drug-tolerant assay (PandA), respectively. For ADA positive patients, other treatment options should be considered depending on the ADA level and its relationship with the clinical threshold value. If the sample is ADA positive with a RECL value >3, the patient should be switched to another treatment. If the ADA level is lower than 3 RECL one could continue treatment but consider repeat testing for ADA again in the coming months to monitor ADA development with the quantitative PandA assay. **Group 3** is within the optimal range of sIFX level and no action is needed if the disease is in remission or demonstrating an acceptable response to treatment. If the disease is not in remission despite sIFX being within optimal range, a drug with another mode of action might be needed. **Group 4** include patients with a sIFX trough level that is higher than the normal range (>6 μg/mL). Lowering the dose should be considered if the patient responds well to infliximab, and other treatment options with another mode of action might be considered for the patient who are not responding well and has active disease.

Based on the results of this study, we suggest a treatment algorithm for interpreting the results of the sIFX and ADA tests (Figure 13). We propose as a first step, measuring the sIFX level, which allows patients to be allocated to one of four groups. **Group 1** and **Group 2** consists of patients with either undetectable infliximab levels (<0.2 μg/mL) or low sub-therapeutic sIFX trough levels (<1 μg/mL) and should therefore be tested for ADA using an ELISA or drug-tolerant assay (PandA), respectively. Not all patients with low sIFX levels can be explained by ADA and an increased infliximab dose could therefore be considered for those found to be ADA negative at this step. For patients found to be ADA positive, other treatment options should be considered depending on the ADA level and its relationship with the clinical threshold value. If the sample is ADA positive with a RECL value >3, the patient should be switched to another treatment due to the likely treatment inhibition and loss of effect owing to ADA. If the ADA level <3 RECL, there may still be an effect of the treatment despite the presence of ADA and it is possible the patient may only be transiently positive. In these cases, one could continue treatment, but consider repeat testing for ADA again in the coming months to monitor for ADA persistence using the quantitative PandA method. **Group 3** includes patients whose sIFX falls within the optimal sIFX range. Therefore, no action is needed if the disease is in remission or demonstrating an acceptable response to treatment. If the disease is not in remission despite sIFX being within optimal range, a drug with another mode of action might be needed. **Group 4** includes patients with a higher than normal trough sIFX level (>6 μg/mL). This might reflect a situation where the drug is not consumed as expected. Therefore, lowering the dose should be considered if the patient is responding well to infliximab. However, treatment options with another mode of action could also be considered for patients who have not responded well and have disease activity. The three exceptional cases with early high ADA titers were excluded from this algorithm and patients such as these, will only be detected if a first tier of the ADA testing is with the PandA method prior to the second infusion. As these patients later developed infusion reactions, one might want to consider changing to an alternative treatment already at this early time point.

## Discussion

Unwanted immunogenicity is a growing challenge for management of patients treated with biopharmaceuticals. To be able to provide the highest quality of care, the consequences of ADA on safety and treatment efficacy have to be addressed. However, the benefit of integrating assessment of drug level and ADA in clinical practice has been questioned for several reasons, including the incomprehensive conclusions of test results with very drug-sensitive ADA assays. Current recommendations advise starting with a screening of drug level and then only testing for ADA in samples with less sIFX than tolerated by the drug sensitive ADA assays (with our in-house ELISA corresponding to <0.2 μg/mL) (28, 34). This is challenged by introducing an ADA assay that is insensitive to drug level. This assay enables reliable ADA testing at any time, not only in trough, but also requires that a new drug level prompting ADA testing is established. Moreover, while there are already existing guidelines for suggested target trough concentrations for sIFX in patients with inflammatory bowel disease (35), the optimal sIFX level in rheumatic diseases is unknown. The aim of this study was to estimate an optimal sIFX range, set a recommendation for when to test for ADA using a drug-tolerant assay and to provide a clinical threshold value for this assay using two large cohorts of infliximab treated patients.

By correlating clinical data with sIFX levels and establishing a suggested optimal range, we found that a substantial proportion of patients were on a suboptimal treatment regimen in a cohort of patients that had been treated for several years. Since sIFX trough levels are highly variable between patients, the initial screening of sIFX trough level is already able to give an indication of treatment efficacy. Collectively, our results from a range of IFX treated diseases in this cohort suggest that a trough sIFX concentration between 1 and 6 μg/mL is possibly associated with better treatment effect and therefore, a potential proxy for an optimal sIFX level. The added value of identifying patients with either too low or too high drug levels is evident, since these two groups have different reasons for their sub-optimal levels which need to be managed in different ways. As of today, although there are several recommendations, there is no consensus on the range of sIFX trough concentration at which patients achieve an optimal therapeutic response. These ranges can also vary depending on what drug, indication and assays are used and therefore, would need to be established for each method (25, 28, 36). As previous studies show, low trough levels of sIFX predict disease activity in RA and as confirmed in this study is most probably due to blocking of the therapeutic effect by ADA (37). These patients might benefit from switching to another TNFi alternative for which the patient has not developed any resistance against. This is in line with the experience with rituximab treatment, where patients developing ADA can regain treatment effect when switching to another anti-CD20 drug (38). Patients with too high drug level were also not doing as well as those within the range of 1–6 μg/mL and therefore it is sensible to analyze these as a separate group. Using the drug tolerant PandA method, we could show that this is not ADA bound in immune complexes. Therefore, the poor treatment effect is likely attributed an initial primary treatment response, and patients have been given a higher dose in attempt to improve this. However, since the drug is not fully consumed before the next infusion, in these patients, the TNF may not be the major driver of disease severity and therefore, may benefit from a therapeutic drug with another mode of action. Similar findings are available for adalimumab treatment (36), where patients with low drug level still had good effect with another TNFi whereas those with a higher drug level did not. There are no biomarkers available that can predict what mode of action the drug should have to achieve optimal treatment response in an individual patient. Indeed, a recent study by Berkhout et al. showed that the level of circulating TNF-α during adalimumab treatment did not predict clinical response in RA and thus cannot be used as a biomarker for treatment discontinuation (39). In practice, clinicians change intervals and dose without having the drug level and ADA information, but this may be optimized with the understanding of how to interpret the drug level and ADA data (40).

One argument against drug level and ADA testing to monitor treatment response in clinical practice is that treatment failure is evident as disease break through. However, in the cross-sectional cohort with >2 years of treatment, we observed that only two-thirds of the patients, at any investigated time points, had the suggested optimal drug concentration (1–6 μg/mL), leaving 30% of the patients with either too low or too high drug levels. For the patients with too low levels (<1 μg/mL), ADA was detected in the majority of serum samples with a drug level below 0.2 μg/mL, meaning that they have been on treatment for a long period without a biologically relevant effect of the drug. ADA was also identified in samples with measurable drug levels using the drug-tolerant assay, but rarely in those with a drug level above 1 μg/mL. Thus, by testing sIFX and ADA in clinical practice, up to one-third of the patients might receive a more optimal treatment. By using PandA for detection of ADA bound in complex with the drug, we identified more ADA positive cases. In addition, this method can be used if one needs to know the ADA status right after infusion, for example to determine if infusion reactions or serum sickness might be due to ADA (41, 42). When comparing the ADA positivity between the two cohorts included in this study, it indeed shows that to some extent ADA positive patients are identified clinically. This is shown, as expected, by a lower frequency of ADA in the cross-sectional cohort (34% of ADA positive samples compare to 23% in the prospective, with sIFX level >0.2 μg/mL), indicating that the ADA positive patients had already been switched to another treatment due to lack of effect determined by clinical outcome. However, a substantial proportion of patients still had suboptimal sIFX level and clinically were not in remission. Thus, measuring the drug level is of clinical value since it can help with the dosing regimen. For example, large individual differences in drug concentration in patients with multiple sclerosis treated with the monoclonal antibody natalizumab has been shown by van Kempen and colleagues were the majority had high trough levels at the time of re-dosing (43). This could partly be explained by that natalizumab is administered at a fixed dose of 300 mg every 4 weeks, not accounting for body weight or pharmacokinetic differences. The authors therefore suggest that the treatment regimen should adapt to a personalized approach to allow efficient use of natalizumab (43).

We investigated the added value of using the bioassay iLite for identification of neutralizing ADA and showed that 66% of the samples were positive for neutralizing ADA. This is far less than what was reported in a study published by Schie and colleagues, which showed that the majority (>90%) of the ADA to infliximab were neutralizing using a TNF competition assay (44). The difference in the number of detected neutralizing samples could be due to assay format, treatment duration at serum sampling, and the small patient cohort size. There was a good correlation between the neutralizing ADA and the PandA RECL values, showing that the level of ADA is of most importance and thus that PandA RECL values can be used as an indicator of neutralizing capacity and clinical effect. Using this approach, it is then most important to know at what level to set the clinical threshold value for ADA. Here we provide an estimate of 3 RECL with the PandA assay as the level of ADA that begins to be detrimental for the therapeutic effect.

The correlation between the PandA assay and the ELISA ADA assay was not perfect, with more discrepancies in the lower levels of ADA positivity. Moreover, the ADA-ELISA is not a quantitative assay and therefore if you want to set a clinical threshold value, then alternative more linear methods are needed. This shows the value of conducting pilot studies in real-life settings to compare assays and to determine the assay specific clinical threshold value before it is applied in the clinic. For the samples with sIFX trough level below 0.2 μg/mL, the ELISA have a higher sensitivity than PandA and this is might be due to the additional washing steps required in the PandA method.

The inverse correlation between drug level and ADA was confirmed. However, five samples with a drug trough level below 0.2 μg/mL that were identified as ADA negative with ELISA were tested with PandA for potential drug/ADA immune complexes, but only one sample showed low ADA reactivity, indicating that other explanations for low drug levels need to be taken into consideration (data not shown). Since infliximab is given intravenously the compliance is controlled for and therefore, could not be the issue here. It is possible that some patients are highly efficient in metabolizing or consuming the drug and studies of biomarkers associated with this trait might resolve this issue.

The timing of the testing is essential. From the prospective longitudinal cohort, we can conclude that the value of testing for sIFX before the initiation period of shorter intervals is over, is questionable. At week 14, the mean sIFX stabilizes, and around half of the ADA positive patients could be identified already here. However, although some of these patients were transiently positive, none of these reached the suggested clinical threshold value of 3 RECL. Some patients that later became ADA positive in ELISA could have be detected as ADA positive with PandA at earlier time points, when the sIFX levels were too high to give reliable ADA test results with ELISA. Thus, if a first-tier screening for sIFX is used, then testing at week 6, before the 3rd infusion with a drug tolerant assay, is suggested to provide the most added value to the clinical practice.

We identified three exceptional cases with high sIFX and high ADA levels for which ADA would have been detected with PandA already at the 2nd or 3rd infusion. Here, the sIFX test would not have guided the decision to test for ADA. At this time point there were no clinical parameters that would have indicated ADA positivity, but all three cases eventually had infusion reactions and secondary treatment failure. A patient with previous infliximab treatment was included in the cohort and this patient also had high ADA identified using the PandA method, already at the 2nd infusion, which would indicate that the three exceptional cases might also have had infliximab treatment before. However, according to the patients files all three were newly diagnosed and had not previously received TNFi treatment prior the start of this study.

Taken together, an infliximab treatment algorithm for RA using the assays included in this study has been be suggested (Figure 13). For patients with low sub-therapeutic sIFX trough levels and no detectable ADA, a dose escalation of infliximab could be beneficial. Patients with therapeutic levels within the recommended range for the chosen endpoint but still an active disease are likely to have pharmacodynamic failure and may benefit from switching to a drug with a different mechanism of action. Samples with a serum trough drug level below 1 μg/mL (to include assay variation around 0.85 μg/mL) would benefit from being screened for ADA using the drug-tolerant assay in order to discriminate whether discontinued treatment or dose escalation should be implemented. Clinical assessment scores would aid the evaluation of patients with a serum drug level above 6 μg/mL (to include assay variation around 6 μg/mL) to ascertain if it could be beneficial to either lower the dose or switch to a drug with a different mechanism of action.

There are some limitations to be mentioned in this study. We have not considered other confounding factors that might increase the risk for ADA. Tatarewicz et al. found that rheumatoid factor, which are present in a majority of RA patients, can interfere with the detection of monoclonal antibodies in immunogenicity assays (45). This is particularly a problem when using a sensitive immunoassay such as the PandA. Tatarewicz and colleagues found that samples from RA patients had a higher baseline value of ADA reactivity than healthy subjects, thus the RF could lead to a false positive signal in the immunoassay (45). Here, we used untreated RA patient serum to set a disease specific cut point, which was slightly higher than normal healthy serum. However, one can question the clinical usefulness of such practice since low positive ADA probably do not have any immediate clinical relevance. Furthermore, the goal of the treatment is to reduce inflammation and thus when this is achieved, the serum profile may be more similar to normal healthy serum.

In conclusion, this cross-sectional and prospective study examined whether measurement of sIFX trough level and ADA testing correlated with treatment response and if adding a drug tolerant assay provided additional clinically useful, accurate and timely ADA results. Decisions of either switching treatment or regulating the dose, need to be guided by evidence based optimal drug levels and a clinical threshold value for ADA. The addition of a drug tolerant assay PandA resolved cases with detectable sIFX and identified ADA positivity earlier than the drug sensitive assay. Even though test results of sIFX and ADA are both heterogeneous and dynamic and thus difficult to interpret on group level, on individual level and as a method to achieve personalized treatment, these data are valuable.

## Data Availability Statement

The raw data supporting the conclusions of this manuscript will be made available by the authors, without undue reservation, to any qualified researcher.

## Ethics Statement

The studies involving human participants were reviewed and approved by Stockholm Regional Ethical Committee (2013/1034-31/3) and Gothenburg Regional Ethical Committee (1028-15, 2016-02-12). The patients/participants provided their written informed consent to participate in this study.

## Author Contributions

All authors listed have made a substantial, direct and intellectual contribution to the work, and approved it for publication.

## Conflict of Interest

CH is currently working at Sanofi Genzyme. MR is currently working at Affybody. AF-H has been supported by an unrestricted research grant from Pfizer (Research Grant Pfizer ref. WI193361) and been given speakers honorary from Pfizer, Biogen, Merck-Serono, and Sanofi-Genzyme. The remaining authors declare that the research was conducted in the absence of any commercial or financial relationships that could be construed as a potential conflict of interest.
