# Supplementary material for: Drug Tolerant Anti-drug Antibody Assay for Infliximab Treatment in Clinical Practice Identifies Positive Cases Earlier
Source: Front Immunol. 2020 Jul 21;11:1365. doi: 10.3389/fimmu.2020.01365 (PMC7385065; doi:10.3389/fimmu.2020.01365)
Supplement: Supplementary file 1 [file Presentation_1.pdf]

Supplementary Material

Drug tolerant anti-drug antibody assay for infliximab treatment in clinical practice identify positive cases earlier

Nastya Kharlamova[1,2,#], Christina Hermanrud[1,2, #], Nicky Dunn[1,2], Malin Ryner[1,2], Karen Hambardzumyan[3], Nancy Vivar Pomiano[3], Per Marits[4], Inger Gjertsson[5], Saedis Saevarsdottir[3], Rille Pullerits[5,6,¤] and Anna Fogdell-Hahn[1,2,¤, *]

[1]Department of Clinical Neuroscience, Karolinska Institutet, Stockholm, Sweden.

[2]Center for Molecular Medicine, Stockholm, Sweden

[3]Rheumatology Division, Department of Medicine, Solna, Karolinska Institutet, Stockholm, Sweden.

[4]Department of Clinical Immunology and Transfusion Medicine, Karolinska University Hospital, Stockholm, Sweden

[5]Department of Rheumatology and Inflammation Research, Institution of Medicine, Sahlgrenska Academy, University of Gothenburg, Gothenburg, Sweden.

[6]Department of Clinical Immunology and Transfusion Medicine, Sahlgrenska University Hospital, Gothenburg, Sweden

[#]Shared first authorship

[¤]Shared senior authorship

*** Correspondence:**
Anna Fogdell-Hahn
Anna.Fogdell-Hahn@ki.se

Table of content

Figure S1. Flow chart of RA patients included in the prospective study.

Figure S2. Infliximab treated patients included in the cross-sectional cohort.

Figure S3. Delta DAS28 levels in a subgroup of the RA patients in the cross-sectional cohort.

Figure S4. Patient Global Assessment in all patients (with available data) treated with infliximab in the cross-sectional cohort.

Figure S5. Trough serum infliximab levels in relation to DAS28 in the cross-sectional cohort after >2 years of infliximab treatment.

Figure S6. Level of trough serum infliximab over time among patients with RA in the prospective cohort.

Figure S7. Incidences and cumulative number of ADA cases over time in the prospective cohort.

Figure S8. EULAR response in relation to anti-drug antibody reactivity in the prospective cohort.

Figure S9. Variability of ADA levels over time.

Figure S10. Receiver operating characteristic curve for setting clinical threshold value of ADA.

Figure S11. Higher levels of ADA seropositive (CCP and/or RF positive) patients compared to seronegative (CCP and RF negative) patients.

Figure S12. ADA levels over time in seropositive (CCP and/or RF positive) RA patients compare to seronegative (CCP and RF negative) patients in the prospective cohort.

Figure S13. ADA levels over time (relative ECL; RECL) in RA patients with rheumatoid factor (RF).

Figure S14. ADA levels over time (relative ECL; RECL) versus smoking status.

Figure S15. Correlation between PandA RECL values and iLite (RLU) results in a subset of ADA positive samples from the cross sectional cohort.

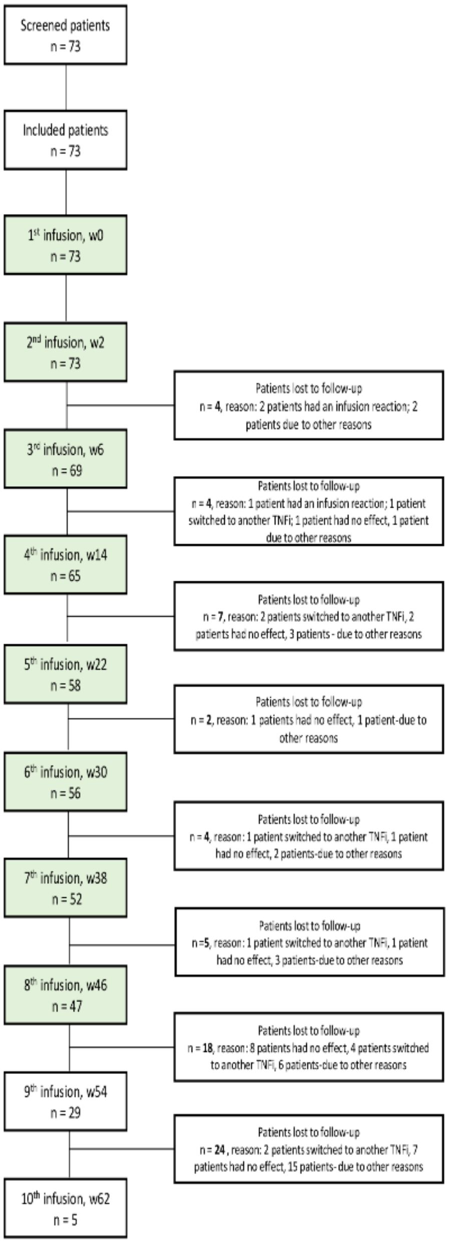

Figure S1. Flow chart of RA patients included in the prospective study. The number of infusions is noted and the approximate time points in weeks. The number and reason for patients lost to follow up are noted. The boxes in green indicate the intended study period (1 year), samples collected after this period from some patients were additional to the original study plan. TNFi = Tumour Necrosis Factor inhibitor.

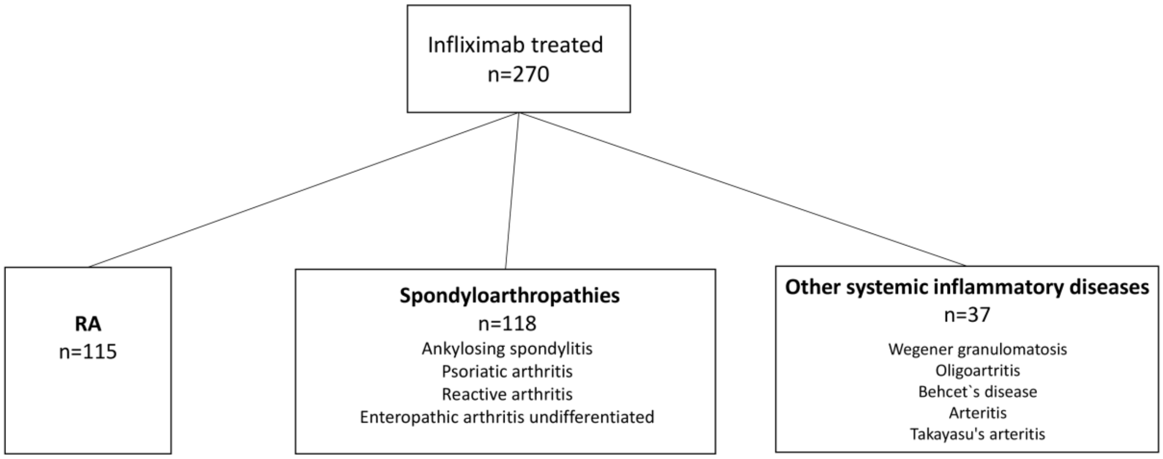

Figure S2. Infliximab treated patients included in the cross-sectional cohort, divided by diagnosis. RA = rheumatoid arthritis

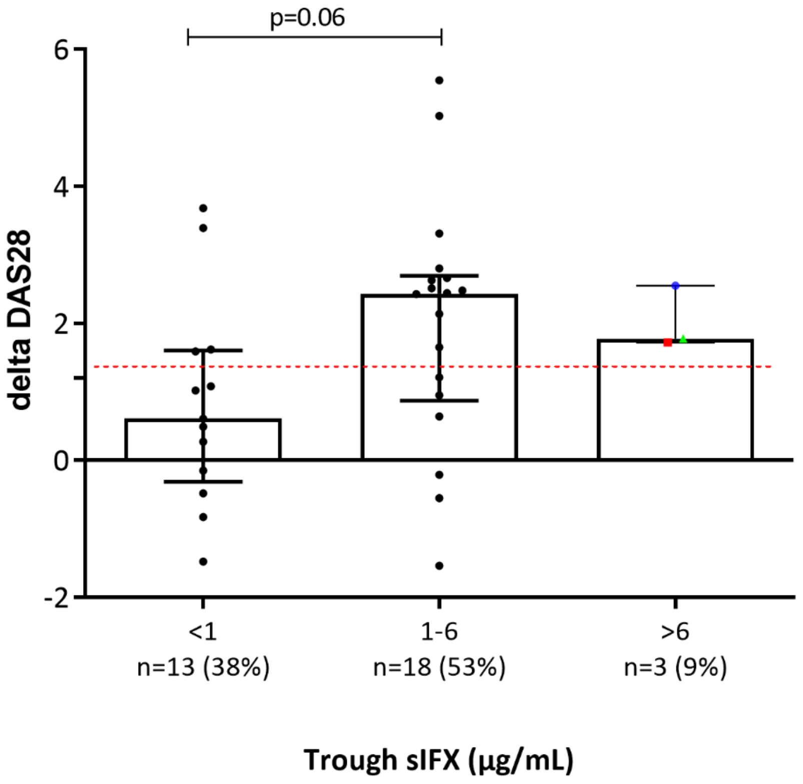

Figure S3. Bar plot of delta DAS28 levels in a subgroup of the RA patients in the cross-sectional cohort (n=34). Patients with RA were divided into three groups based on trough sIFX: under 1 µg/mL, 1-6 µg/mL, and above 6 µg/mL. Delta DAS28 was notably worse in patients with a serum infliximab (sIFX) trough level below 1 µg/mL compared with those with a drug level between 1-6 µg/mL. Dotted line depicts delta DAS28 of 1.2 and indicates good response bellow this line. The trough sIFX levels presented as median (bars) and interquartile range (whickers). Colored dots represent 3 RA patients.

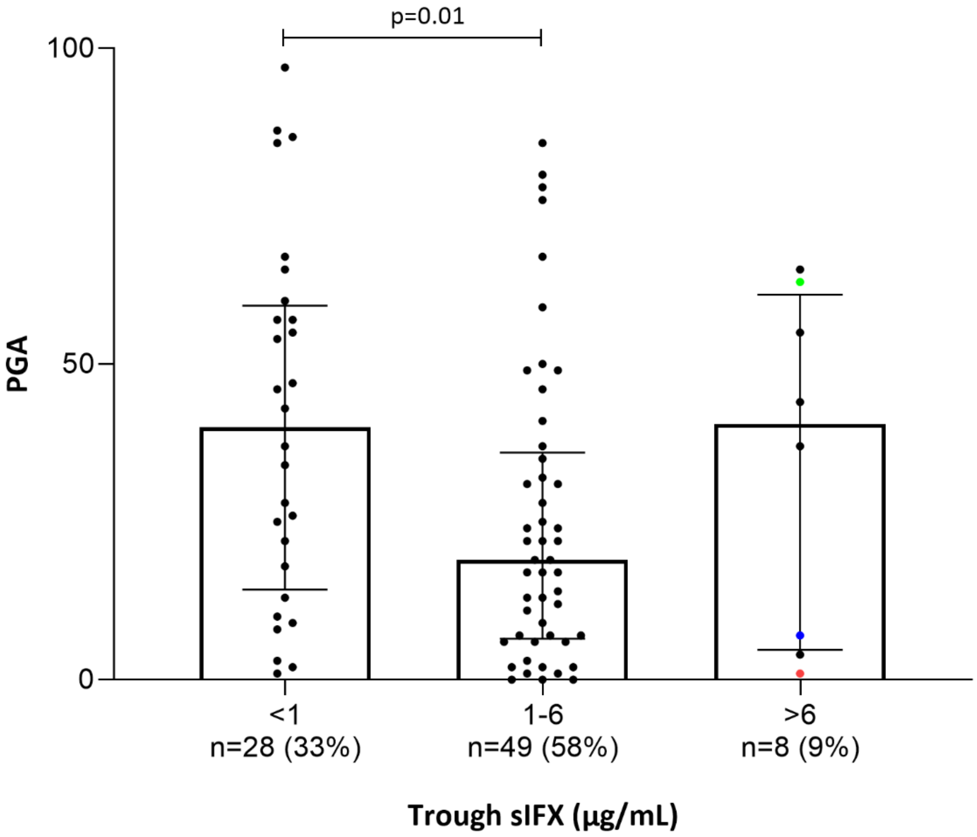

Figure S4. Bar plot of the levels of Patient Global Assessment (PGA) in all patients (with available data) treated with infliximab in the cross-sectional cohort. Patients were divided into three groups based on trough serum infliximab (sIFX): under 1 µg/mL, 1-6 µg/mL, and above 6 µg/mL. PGA was significantly higher in patients with a sIFX trough level below 1 µg/mL compared with those patients who had an optimal drug level. The trough sIFX levels presented as median (bars) and interquartile range (whickers). Colored dots represent 3 RA patients.

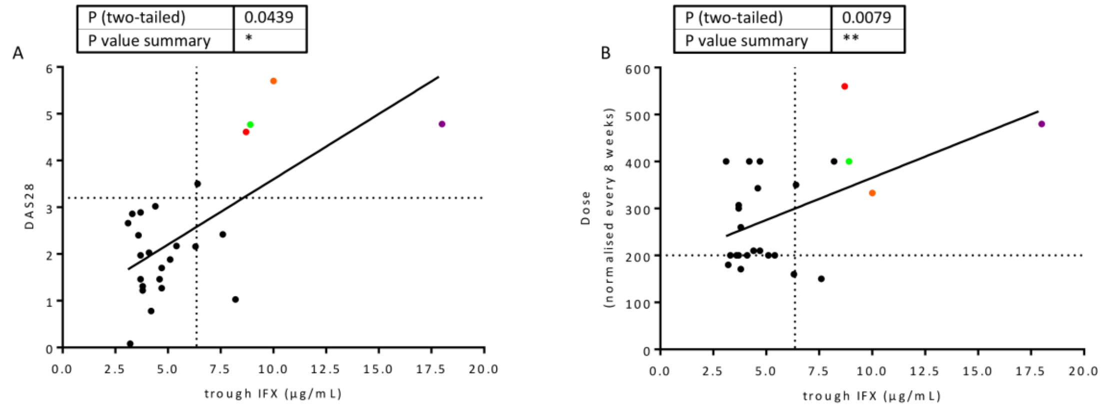

Figure S5. Trough serum infliximab levels in relation to DAS28 in the cross-sectional cohort after >2 years of infliximab treatment (A) and normalized to dose at 8-week intervals. (B). Patients with high serum infliximab levels have higher DAS28 levels and higher dose of infliximab (when normalized to dose at 8 weeks interval).

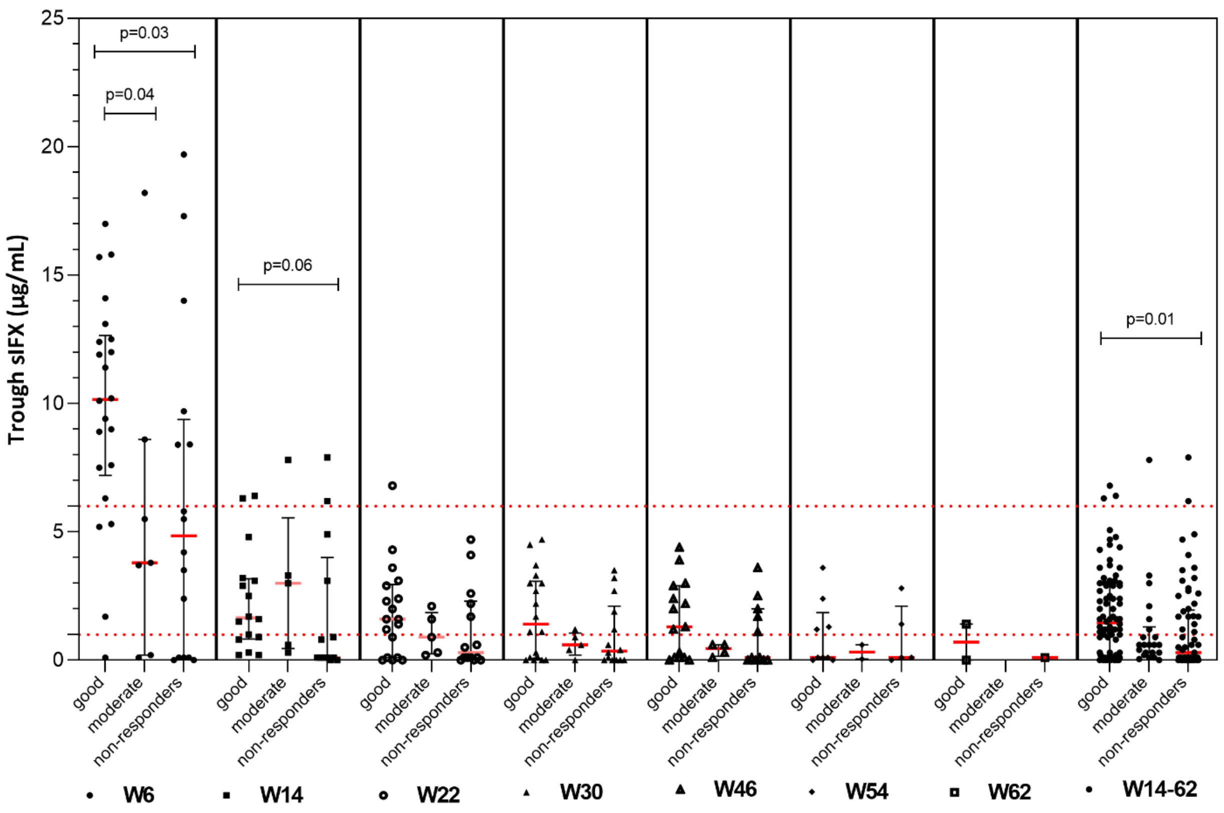

Figure S6. Level of trough serum infliximab (sIFX) over time among patients with RA in the prospective cohort. Patients were divided on good-, moderate- and non-responders based on EULAR criteria. The dots represent each individual patient. Level of trough sIFX varied between time points, but all together the non-responders had a significantly lower sIFX level than the good responders (week 14-62). The trough sIFX levels presented as median (red line) and interquartile range (whickers). The dotted lines represent the lower limit of 1µg/mL and upper limit (6 µg/mL) for optimal therapeutic effect of sIFX. W, week

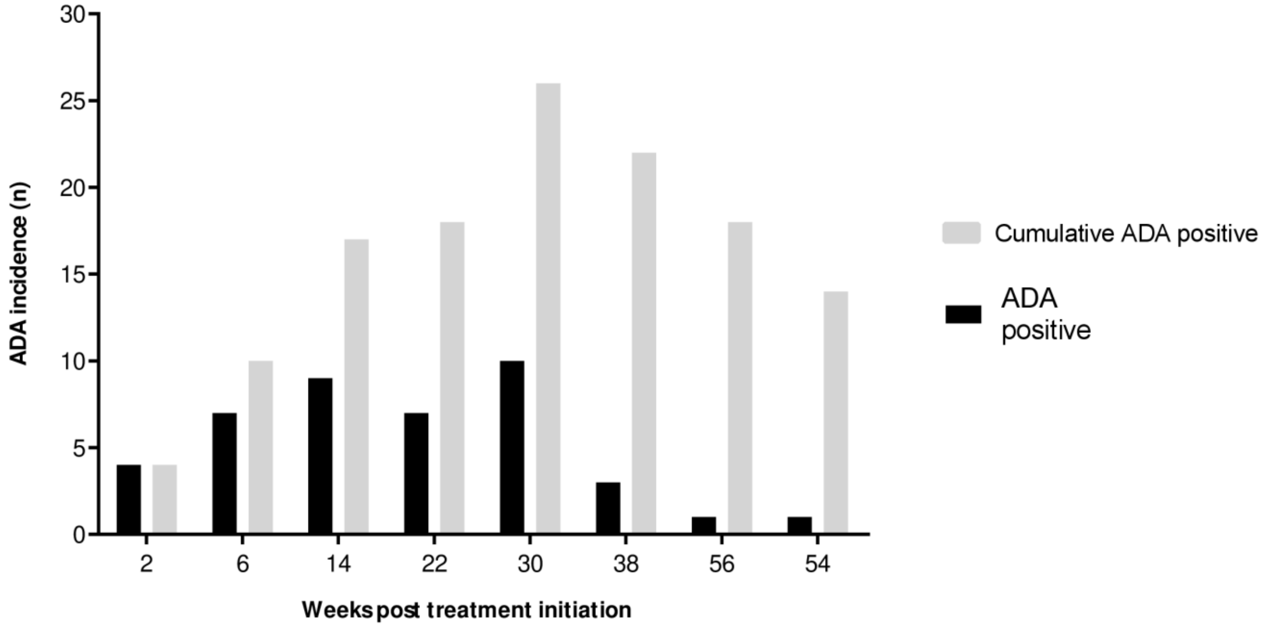

weeks	2	6	14	22	30	38	46	54
total number of patients	73	69	65	58	56	52	47	29
% of accumulated ADA positive patients	5,48	14,49	26,15	31,03	46,43	42,31	38,3	48,28

Figure S7. A histogram representing the incidences and cumulative number of ADA cases over time in the prospective cohort. ADA development was determined using the drug-tolerant PandA assay. ADA was found to develop early after treatment initiation and the first positive samples was identified prior to the 2nd infusion at week 2. The incidence of ADA increased until the 6th infusion at week 30, but patients with newly positive samples were detected until the 9th infusion week 54. The cumulative prevalence of ADA increased up to the 6th infusion at week 30. ADA = anti-drug antibodies

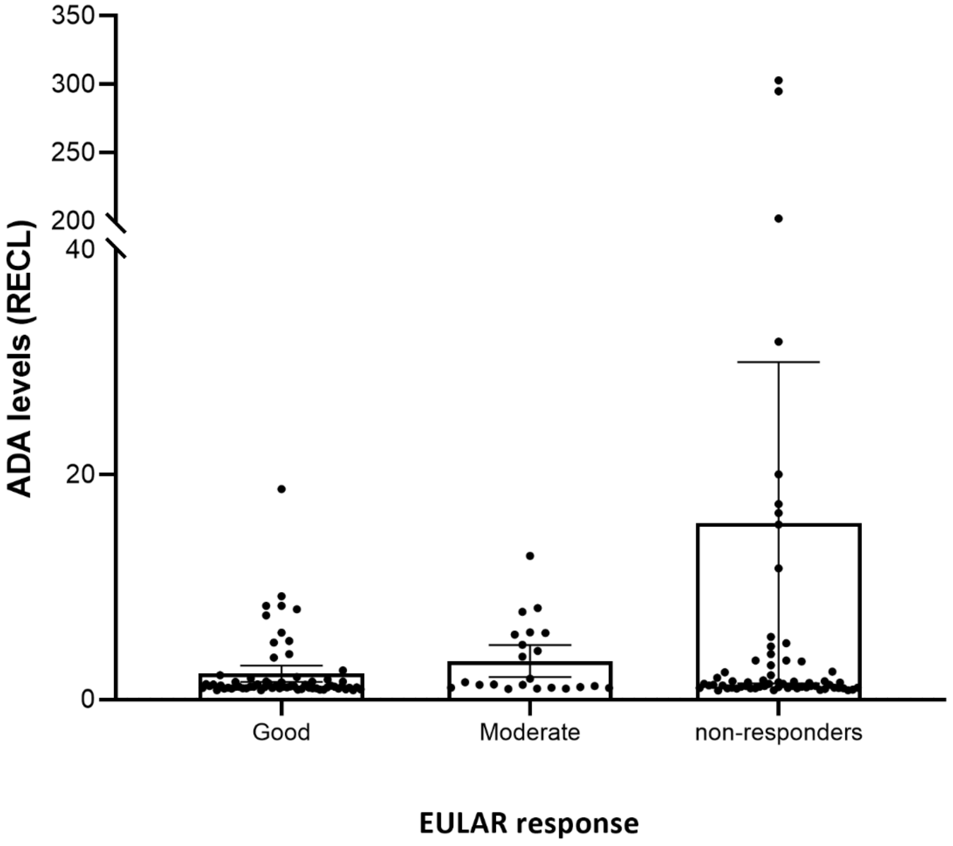

Figure S8. EULAR response in relation to anti-drug antibody reactivity (relative ECL; RECL) in the prospective cohort. Patients were stratified based on EULAR response categories as good, moderate or non-responder. A higher mean ADA value was found in the group with EULAR non-responders.

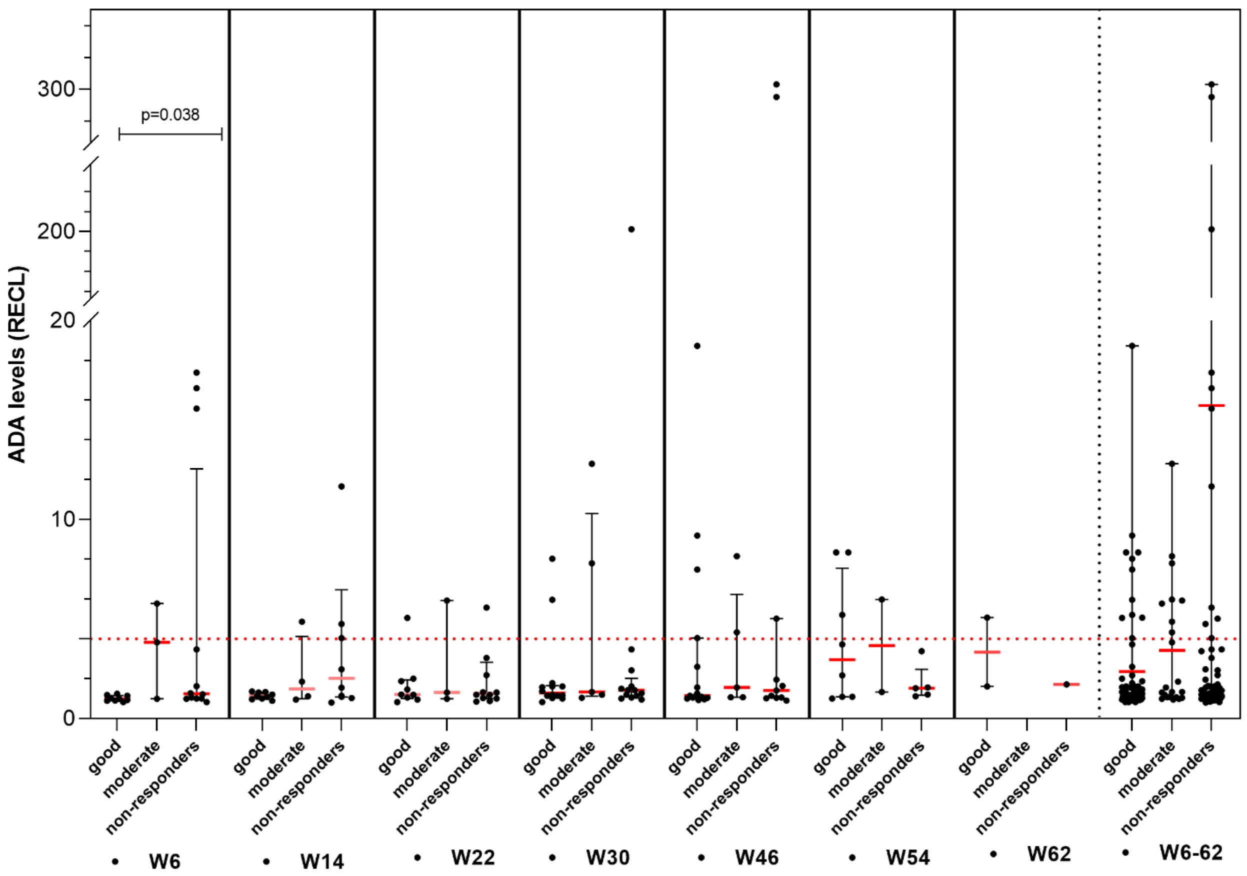

Figure S9. Level of ADA (relative ECL; RECL) variability over different time points. Overall the non-responders had a higher ADA level than the good responders (week 6-62) in prospective cohort. Patients were divided on good-, moderate- and non-responders based on EULAR criteria. The dots represent each individual patient. The dotted line represents a clinical threshold value of 3.25 for ADA. W, week

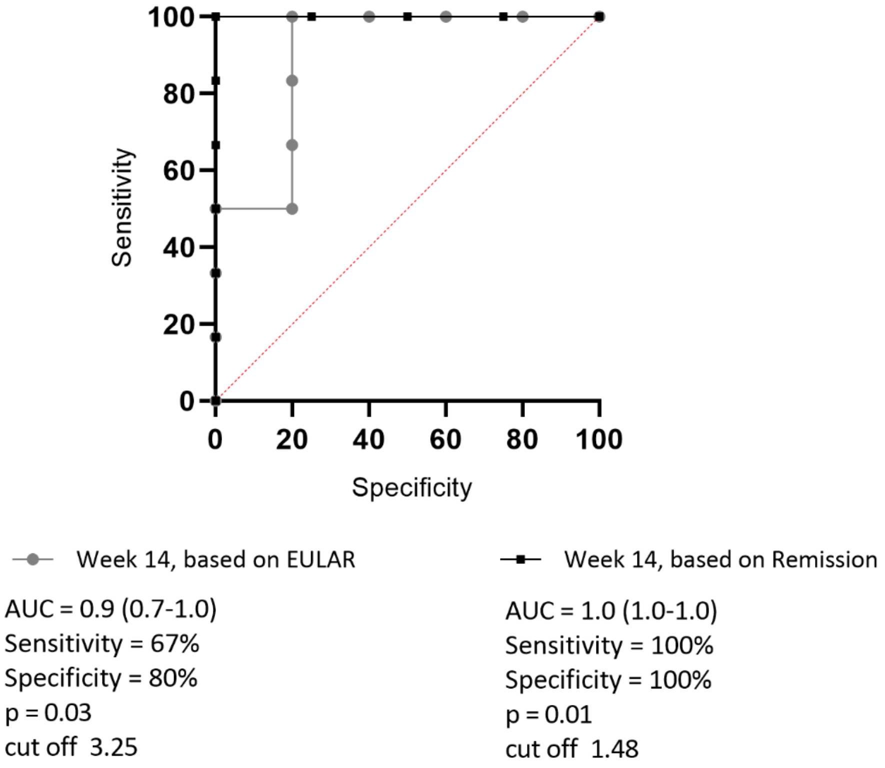

Figure S10. Receiver operating characteristic curve (ROC) for setting clinical threshold value of ADA. ADA levels for ROC curve were taken at week 14 after treatment initiation from the prospective cohort. Black line with quadrates shows the curve based on Remission criteria. Grey dotted line represents the curve build taking into account EULAR criteria (good and non-responders). A clinical threshold value of 3.25 (AUC 0.9, p=0.028) were set based on EULAR criteria. When the remission state was taken as an outcome the clinical threshold value was 1.48 (AUC 1.0, p=0.01).

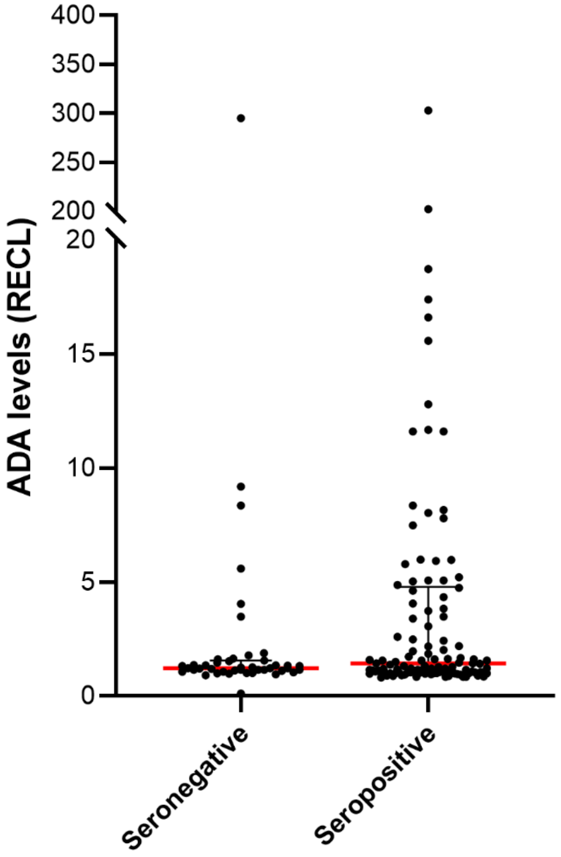

Figure S11. Higher levels of ADA were detected in samples (n=114) from seropositive (CCP and/or RF positive) patients compared to the samples (n=43) from seronegative (CCP and RF negative) patients. Data was retrieved from prospective cohort. ADA levels presented as median (red lines) and interquartile range (whickers).

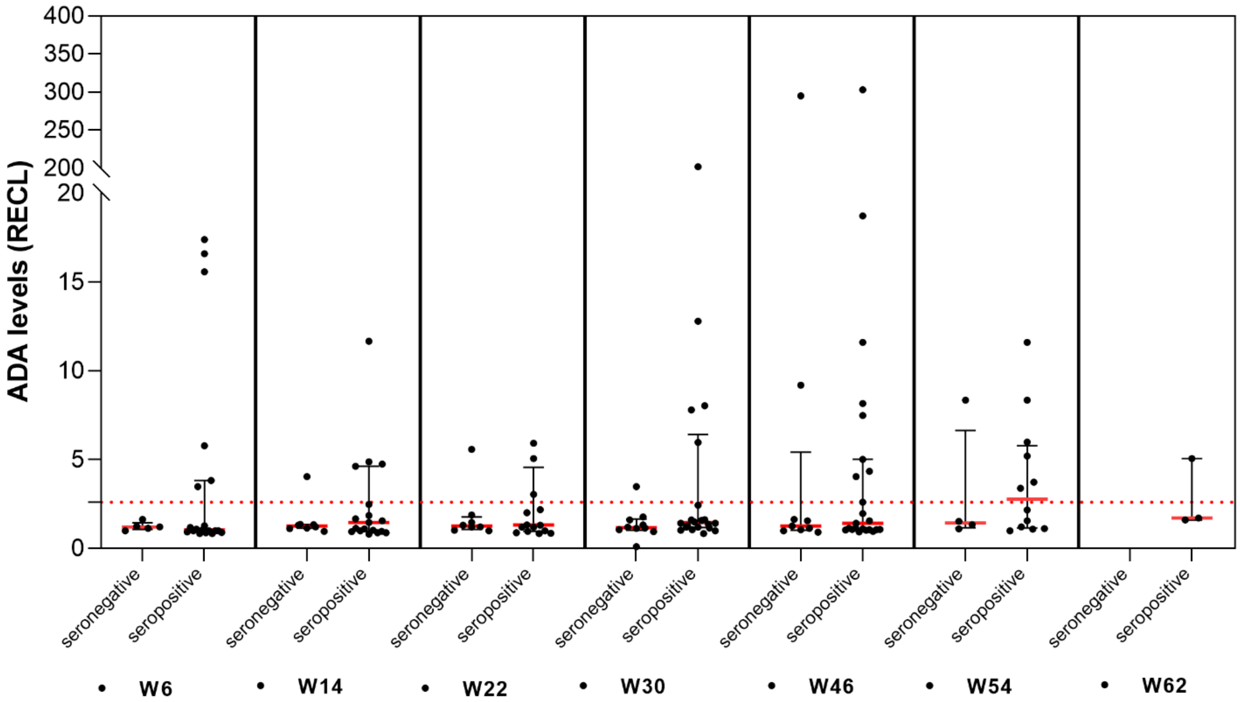

Figure S12. Higher levels of ADA over time (relative ECL; RECL) were detected in seropositive (CCP and/or RF positive) RA patients compare to seronegative (CCP and RF negative) patients in the prospective cohort. The dots represent each individual patient. The dotted line represents the confirmed a clinical threshold value of 3.25 for ADA. W, week

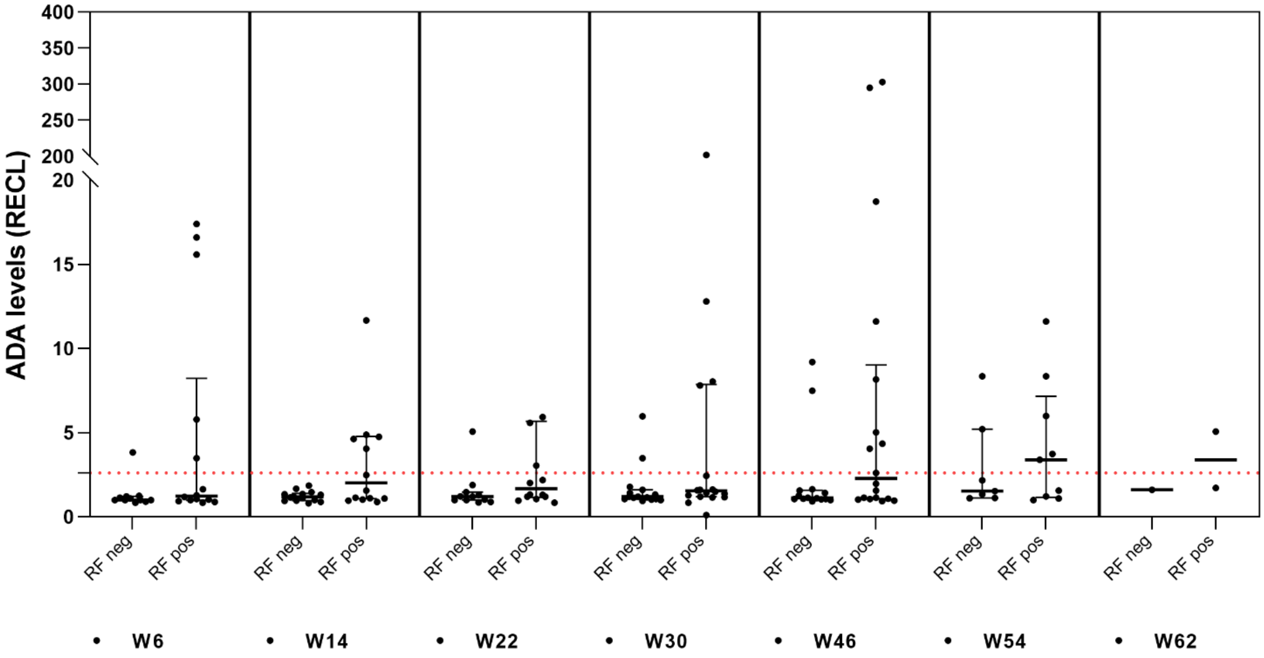

Figure S13. Higher levels of ADA over time (relative ECL; RECL) were detected in RA patients with rheumatoid factor (RF) compare to the patients who were negative for RF in the prospective cohort. The dots represent each individual patient. The dotted line represents a clinical threshold value of 3.25 for ADA. W, week

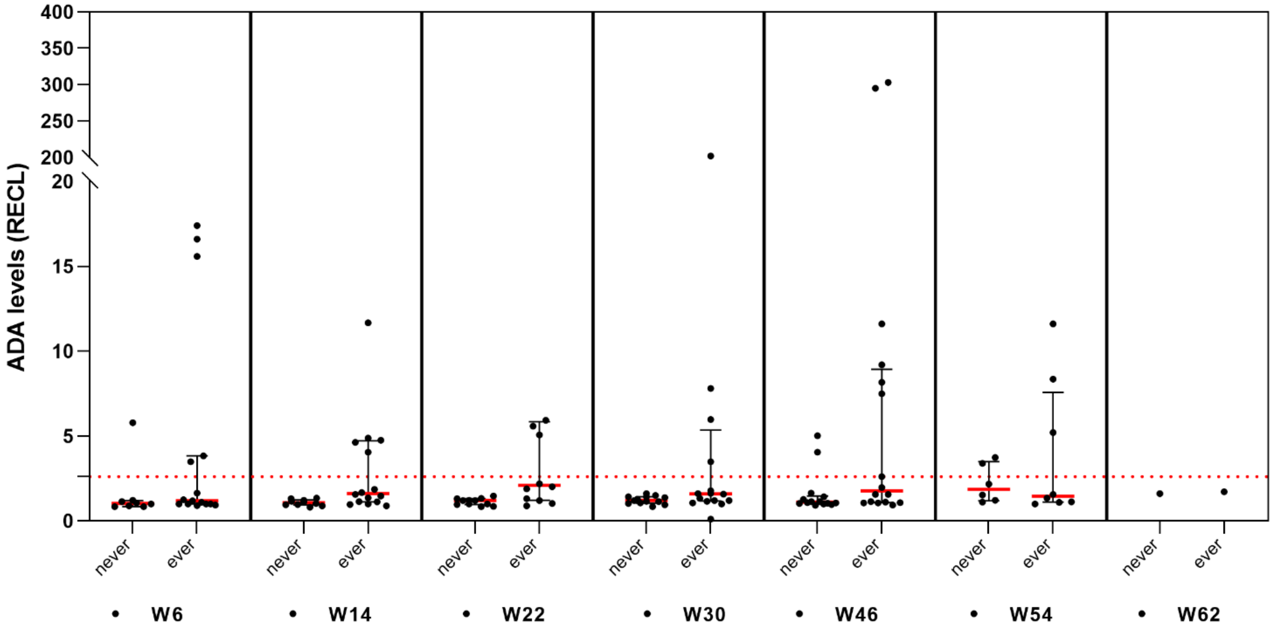

Figure S14. Higher levels of ADA over time (relative ECL; RECL) were detected in ever smokers compare to the patients who had never smoked. The data was retrieved from the prospective cohort. The dots represent each individual patient. The dotted line represents a clinical threshold value of 3.25 for ADA. The ADA levels presented as median (red lines) and interquartile range (whickers).

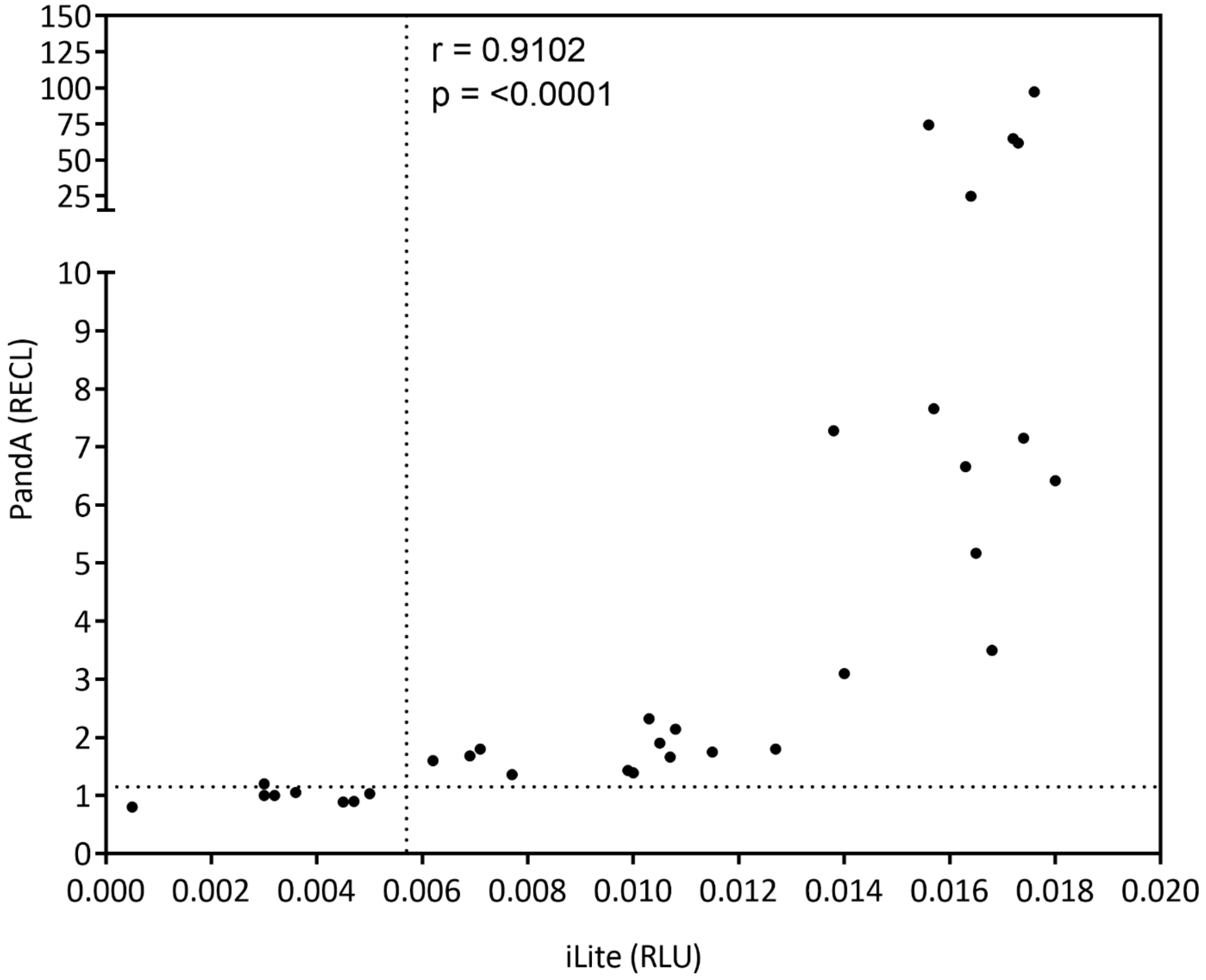

Figure S15. Graph demonstrating the correlation between PandA RECL values and iLite (RLU) results in a subset of ADA positive samples from the cross sectional cohort (n = 29). The dot plot is showing the spread of TNF alpha activity (CPS) in a subset of ADA positive samples from the cross-sectional cohort.
